# Aperiodic and Periodic Components of Ongoing Oscillatory Brain Dynamics Link Distinct Functional Aspects of Cognition across Adult Lifespan

**DOI:** 10.1523/ENEURO.0224-21.2021

**Published:** 2021-10-15

**Authors:** Kusum Thuwal, Arpan Banerjee, Dipanjan Roy

**Affiliations:** Cognitive Brain Dynamics Lab, National Brain Research Centre, Manesar, Gurgaon, Haryana 122052, India

**Keywords:** 1/f activity, band ratio, healthy ageing, peak frequency, self-organized critical state, visual short-term memory

## Abstract

Signal transmission in the brain propagates via distinct oscillatory frequency bands but the aperiodic component, 1/f activity, almost always co-exists which most of the previous studies have not sufficiently taken into consideration. We used a recently proposed parameterization model that delimits the oscillatory and aperiodic components of neural dynamics on lifespan aging data collected from human participants using magnetoencephalography (MEG). Since healthy aging underlines an enormous change in local tissue properties, any systematic relationship of 1/f activity would highlight their impact on the self-organized critical functional states. Furthermore, we have used patterns of correlation between aperiodic background and metrics of behavior to understand the domain general effects of 1/f activity. We suggest that age-associated global change in 1/f baseline alters the functional critical states of the brain affecting the global information processing impacting critically all aspects of cognition, e.g., metacognitive awareness, speed of retrieval of memory, cognitive load, and accuracy of recall through adult lifespan. This alteration in 1/f crucially impacts the oscillatory features peak frequency (PF) and band power ratio, which relates to more local processing and selective functional aspects of cognitive processing during the visual short-term memory (VSTM) task. In summary, this study leveraging on big lifespan data for the first time tracks the cross-sectional lifespan-associated periodic and aperiodic dynamical changes in the resting state to demonstrate how normative patterns of 1/f activity, PF, and band ratio (BR) measures provide distinct functional insights about the cognitive decline through adult lifespan.

## Significance Statement

Ageing is accompanied by the decline in cognitive functions and age itself is a major risk factor for Alzheimer’s disease and other neurologic conditions. Our study provides magnetoencephalogram (MEG) 1/f aperiodic and periodic markers across the healthy adult lifespan and shows that different frequency bands and their spectral features (aperiodic and periodic component) mediate age-related changes across different brain regions, in multiple cognitive and metacognitive domains, which not only provides us with a better understanding of the aging process but would also help in better prevention of cognitive impairments. A clear characterization of the association between baseline oscillatory component, 1/f activity, band ratio (BR), healthy aging and cognition, is established in this study.

## Introduction

Spontaneous brain dynamics indexed by variation of neuroelectromagnetic potential may reflect the local as well global change associated with healthy aging processes that guide behavioral response through lifespan (Bishop et al., 2010; Foster et al., 2016; Sahoo et al., 2020). While the periodic component of spontaneous activity has been extensively studied as an objective measure for cognitive phenotyping (Klimesch, 1999; Knyazev, 2007; Schutter and Knyazev, 2012), the non-oscillatory/aperiodic background component also known as “1/f” activity received less attention compared with the former. This spontaneous 1/f aperiodic brain dynamics almost always pervasively co-exists in EEG/magnetoencephalography (MEG)/LFP signals (Lyne et al., 1992; Linkenkaer-Hansen et al., 2001; Beggs and Plenz, 2003; Bédard et al., 2006; Voytek et al., 2015) and critically influence oscillatory signatures in the context of healthy aging and constrain task performance (Voytek et al., 2015; Donoghue et al., 2020). As we age, we are faced with the likelihood that our cognitive faculties will decline for example, ascertain memory (Craik, 1994; Ouyang et al., 2004; Nyberg et al., 2012), shift of sustained attention (Gazzaley et al., 2005), and processing speed (Gazzaley et al., 2007; Salthouse, 2010). There were recent attempts to relate 1/f activity, where it is being considered as a marker of “neural noise,” with N900 lexical prediction (Dave et al., 2017), working memory (Donoghue et al., 2020), and grammar learning (Cross et al., 2020).

The existence of ubiquitous 1/f activity in neuronal systems organized across various spatial scales is one of the key features of signal variability and collectively referred to as “noise.” However, a recent perspective suggests a serious reconceptualization is necessary to define what constitutes the “signal of interest” or noise in neuroscience (Uddin, 2020). The characteristics of 1/f component, slope and offset, of the ongoing oscillatory power has been found to be dynamic in nature. One possible mechanism for this dynamical change is an increased baseline activity (Voytek et al., 2015) or at a more fundamental level, a phenomenon called self-organized criticality (Bak et al., 1987). In neuroscience, the presence of critical brain states can shape an organism’s ability to optimally switch between task states (Buzsáki, 2006). However, some studies have postulated that the origin of 1/f may lie in the tissue properties (Bédard et al., 2006). Therefore, we hypothesized that aging is consonant with alteration of physiological properties in brain tissue, so any changes along aging dimension in 1/f owes its origin to the tissue properties. Subsequently, we propose that prospective age-corrected correlations between 1/f and behavioral performance in tasks will reveal whether the tissue properties can alter the self-organized criticality of functional brain states and subserve as a non-specific mediator of behavior and cognitive functions.

Other than the characteristics of 1/f component, slope and offset, physiological aging has been further characterized by progressive change in oscillatory power, central frequency (CF), and functional connectivity (Voytek et al., 2015; Murty et al., 2020; Sahoo et al., 2020; Tran and Reddy, 2020). For example, a consistent line of research associated higher individual peak α frequency (PAF) across adulthood with better working memory and better reading comprehension (Klimesch, 1999; Clark, 2004). There are also noticeable discrepancies among numerous existing aging studies based on the estimation of difference of power between younger and elderly individuals in the frequency band of interest (Klass and Brenner, 1995; Aurlien et al., 2004; Cummins and Finnigan, 2007; Stomrud et al., 2010; Ishii et al., 2017; Scally et al., 2018; Sahoo et al., 2020). Hence, it is often notoriously difficult to reconcile those age-associated oscillatory findings during spontaneous activity and trusting power changes in the relevant frequency band were estimated accurately. One possible reason for this inconsistency might be the mixing of oscillatory power with the aperiodic background 1/f activity, which was not taken into sufficient consideration by most of the studies.

On the other hand, frequency band ratio (BR) is a common measure in investigating attention deficit hyperactivity disorder (ADHD; Lubar, 1991; Snyder and Hall, 2006; Arns et al., 2014), executive functioning (Lubar, 1991; Angelidis et al., 2016; Gordon et al., 2018; van Son et al., 2019) and working memory capacity (Moran et al., 2010). We propose here that both shifts in individual peak frequency (PF) and BR index clearly different aspects of functional changes associated with aging impacting short-term working memory speed and accuracy of retrieval on one hand and cognitive capacity on the other.

Together, we suggest the three different measures, 1/f slope-offset reflecting aperiodic activity, PF and BR reflecting periodic activity provide distinct functional insights about the neural underpinning of the healthy aging process.

## Materials and Methods

### Participants

The Cambridge Centre for Ageing and Neuroscience (Cam-CAN) is a large-scale, multimodal, cross-sectional adult lifespan (18–88) population-based study. The Cam-CAN consists of two stages. In stage 1, 2681 participants had gone through general cognitive assessments at their home. Tests for hearing, vision, balance, and speeded response were also assessed. Additionally, measures taken in stage 1 served to screen participants for stage 2. Those with poor hearing, poor vision, with neurologic diseases such as stroke, epilepsy, or a score <25 in MMSE (cognitive assessment examination) were excluded from the further participation. From stage 1 to stage 2, 700 participants were screened (50 men and 50 women from each age band). All screened participants were recruited for testing at the Medical Research Council (United Kingdom) Cognition and Brain Sciences Unit (MRC-CBSU) in Cambridge. In this stage, MRI scans, MEG recordings, and cognitive task data were collected, all the participants performed a range of psychological tests and neuroimaging assessments, but only the MEG RS data and visual short-term memory (VSTM) task data are included in this study. Out of 700 participants, MEG data from 650 subjects were available. Age values of participants were divided into four age groups for categorical analysis (see statistical analysis): young adults (YA), middle elderly (ME), middle-late (ML), older adults (OA). Earlier studies have done similar grouping (Chan et al., 2014; Sahoo et al., 2020). A total of 70 participants were randomly chosen from each age group resulting in a total of *N* = 280 subjects comprising all four important stages of adult lifespan (Table 1).

**Table 1:** Each representative age is divided into four groups YA, ME, ML, and OA

Serial Number (S.No)	Group	Age	*N*
1	Young adults (YA)	18–35	70
2	Middle elderly (ME)	36–50	70
3	Middle-late (ML)	51–65	70
4	Old adults (OA)	66–88	70

### Data acquisition

#### MEG resting-state data

MEG Data used for this study were obtained from the Cam-CAN repository (available at http://www.mrc-cbu.cam.ac.uk/datasets/camcan/; Taylor et al., 2017; Shafto et al., 2014). For all the 700 participants, MEG data were collected by Elekta Neuromag, Helsinki at MRC-CBSU using 306 channels, consisting of 102 magnetometers and 204 orthogonal planar gradiometers. MEG data collection was done in a light magnetically shielded room (MSR). A high pass filter of 0.03-Hz cut-off was used to sample the data at 1000 Hz. Head-position indicator (HPI) coils were used to continuously assess the head position within the MEG helmet. To monitor blinks and eye movements, two pairs of bipolar electrodes were used to record horizontal and vertical electrooculogram signals. To monitor pulse-related artefacts, one pair of electrodes was used to record electrocardiogram signals. MEG data collected for resting state required the participants to sit still for a minimum of 8 min and 40 s with their eyes closed. From this subset, 280 participants were included in the present study (70 in each group).

#### VSTM stimuli and task

**Figure 1.**
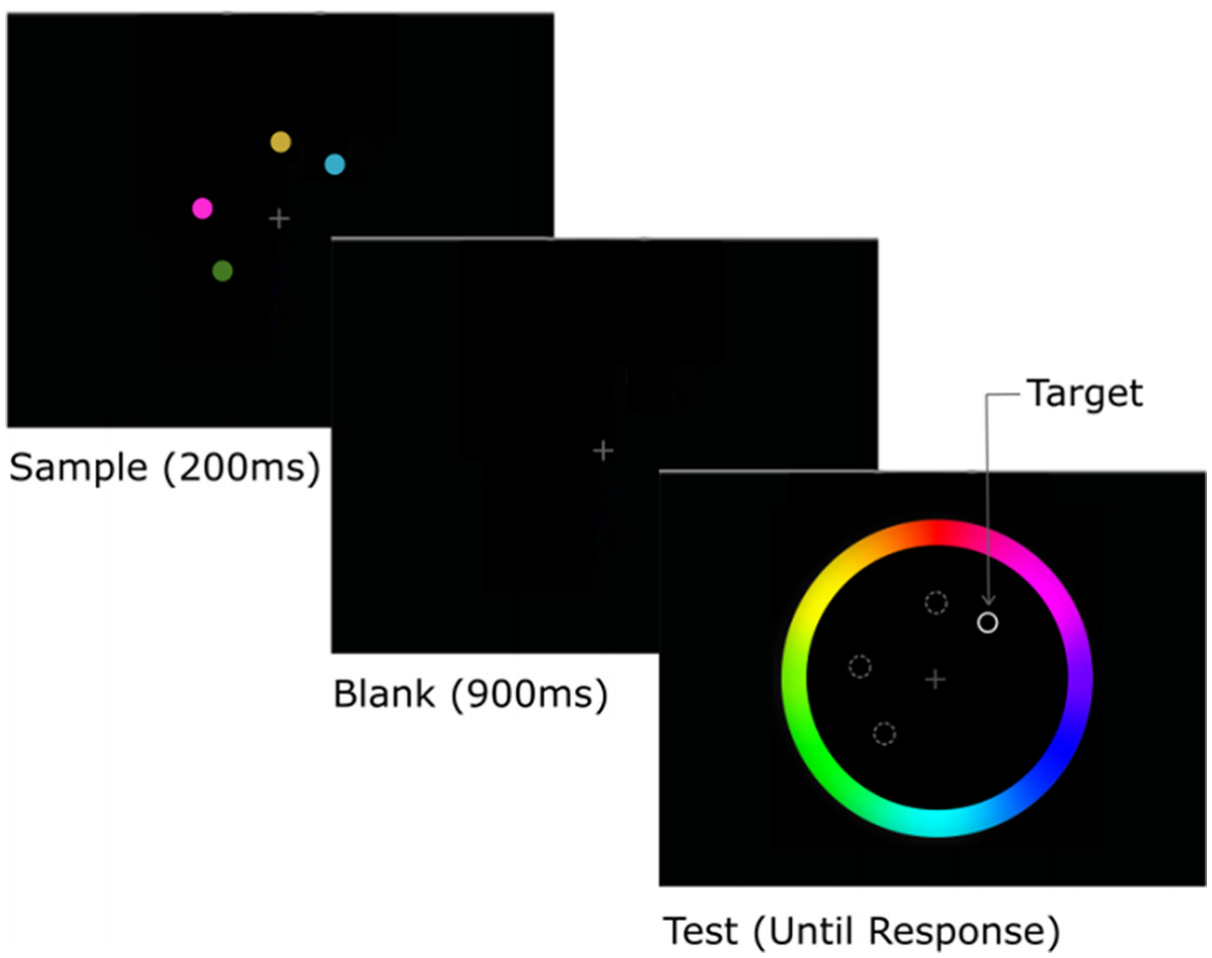
Experimental design of the color recall task. Example trial, with memory load of four items (data were taken from Cam-CAN repository; adapted from Mitchell et al., 2018).

In Cam-CAN, the design was adapted from Zhang and Luck (2008; Fig. 1). On each trial, participants were presented with one, two, three, or four colored discs (mimicking different memory load conditions) for 250 ms. Following that, a blank screen was presented for 900 ms to hold those colors in memory. One of the original locations was highlighted by a thick black border (acting as a probe for participants to remember the color at that location), and at the same time, a response color wheel was presented. Participants had as much time as required to report by touching or clicking, as accurately as possible the remembered hue of the highlighted disk. No feedback was given. After every trial, there was a 830-ms fixation period. Participants complete two blocks of 112 trials, with memory load (1, 2, 3, or 4) counterbalanced and randomly intermixed. For each set size (memory load), the following measures (Table 2) were estimated by fitting the error distribution with a mixture model of von Mises and uniform distributions, proposed by Zhang and Luck (2008) and modified by Bays and Husain (2008; for detailed analysis, see Zhang and Luck, 2008; Mitchell et al., 2018). In brief, as a model-free index of performance, the response error, the angular difference between the target color presented and the color reported, was calculated. This model-free index cannot be used to distinguish errors because of imprecise memory of an item, from errors because of reporting the wrong item, or guessing when an item is not kept in memory at all. To estimate these, Maximum likelihood estimation was used to decompose the data from each subject into three parameters that represent a mixture of a uniform distribution of errors and a von Mises distribution of errors. The von Mises distribution is the circular analog of the Gaussian distribution and was used because the tested color space was circular. The uniform distribution was represented by a single parameter, *P_m_*, which is the probability that the probed item was present in memory at the time of the probe. *K* is calculated by multiplying the memory load by *P_m_*. The von Mises distribution was represented by two parameters, its mean (μ) and its Standard Deviation (SD); μ reflects any systematic shift of the distribution away from the original color value. The “precision” of each item held in memory is reported as the reciprocal of the SD of the fitted von Mises distribution. Subjects indicated their uncertainty in their choice of color by the length of time they touched the wheel: as they held their finger down, white confidence intervals spread out around the selected point indicating greater uncertainty about their selection. To assess metacognitive awareness, the angular width of the reported confidence intervals provided a trial-by-trial measure of subjective uncertainty. To summarize overall uncertainty for each individual and condition, the mean was taken across trials. Participants with smaller values thus reported more confidence in their responses.

**Table 2:** Estimated behavioral measures of VSTM task

Serial Number (S.No)	Variable	Description
1	Precision	Accuracy of reportable items (degrees^−1^)
2	RT	Median reaction time (ms)
3	K(VSTM capacity)	Number of reportable items (k-score)
4	Mean uncertainty	Size of confidence interval within which answer is thought to lie (degrees)

#### MEG data preprocessing

MEG processed data were provided by Cam-CAN. Preprocessing pipeline included temporal signal space separation, applied on continuous MEG data to remove noise from the HPI coils, environmental sources and continuous head motion correction. For removing the main frequency noise (50-Hz notch filter) and to reconstruct any noisy channel, max filter was used. More details about data acquisition and preprocessing have been presented elsewhere (Shafto et al., 2014; Taylor et al., 2017). Additionally, we performed independent component analysis (ICA) to get rid of the artifacts and removal of higher order harmonics present in different frequency bands in the signal following previous studies (Taylor et al., 2017; Sahoo et al., 2020).

#### Data analysis

**Figure 2.**
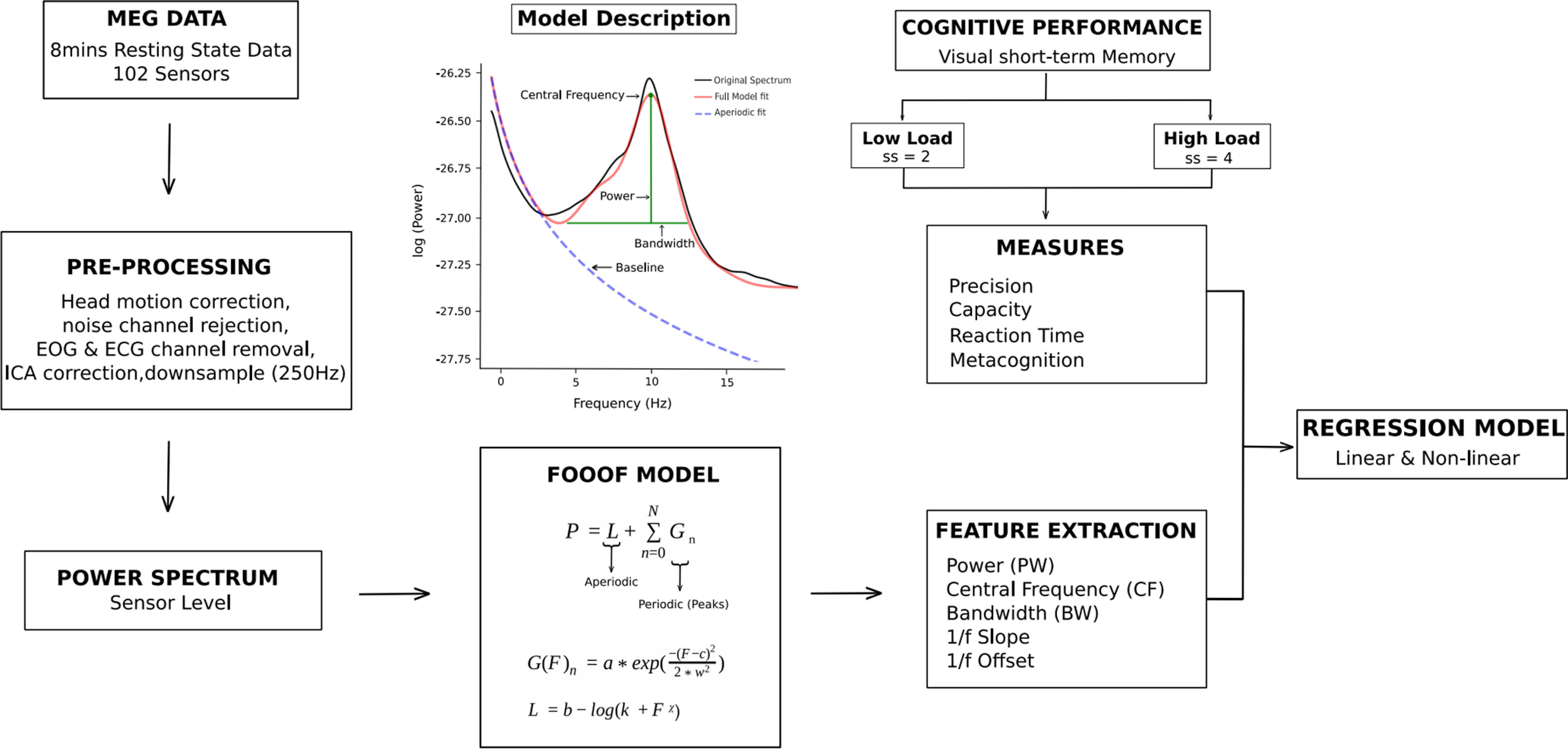
Data processing and analyses pipeline.

We analyzed the MEG and behavioral data in MATLAB and python using custom made scripts. In this study, we used Python MNE for preprocessing; standard python libraries including *Scipy*, *Pandas*, and *NumPy* for data management and processing; and python-*matplotlib* and *seaborn* for data visualization. The analysis pipeline is presented in Figure 2.

##### Power spectral density (PSD) using Welch’s periodogram method

The Power spectrum *S_xx_(f)* of a signal *x(t)* capture how the strength of the signal is distributed in the frequency domain. Using fast Fourier transform (FFT; a variant of Fourier analysis), the representation of raw signal (time or space) is transformed into a frequency representation of the signal. Processed MEG data provided in .fif format was analyzed using Fieldtrip toolbox (Oostenveld et al., 2011). Data for each *N* = 280 subjects were first downsampled from 1000 to 250 Hz. The frequency resolution was held at 0.05 Hz. PSD was estimated using Welch’s periodogram method (*pwelch* function) implemented in MATLAB 2019b. For each participant, 102 magnetometer sensor’s time series data resulted in a matrix of size 102XT, where Tcorrespond to the number of time points. Each sensor’s $c^{\prime }s$ time series *x*_c_(*t*) was further divided into segments of 20 s (epochs) without any overlap. Spectrum was estimated for each segment after multiplying the time series segment with a Hanning window. We estimated a global spectrum, representative of each subject, i.e., *S*_I_(*f*) by taking a grand average of spectrums across all 102 magnetometer channels.

(1) $$S_{I}\left(f\right)=\underset{c}{\sum }s_{I}\left(c,f\right)$$

For each participant, resulted power spectrum matrix was νXc. For group-wise analysis, each participant’s spectrum was averaged across sensors of interest.

##### Extracting periodic and aperiodic features using a parameterization model

To separate the periodic (oscillatory) component from the aperiodic component of the signal power spectra, we used a recently proposed parameterization model, fitting-oscillations-and-one-over-f (FOOOF toolbox; for full description, see Donoghue et al., 2020). In brief, the PSDs calculated using *pwelch* were given as an input to the FOOOF model, which considers PSDs as a linear sum of aperiodic 1/f like characteristics of neural power, and it is entirely described by the aperiodic “exponent” and “offset.” Periodic components describe putative oscillations that describe power above aperiodic component [so-called “peaks,” simulated as Gaussian function; are described by PF in Hertz, peak power over and above the 1/f signal in arbitrary units (au) and bandwidth (BW; Hz)]. Larger BW in given frequency band indicates the deviation of power from the baseline and the spread across a wider frequency range. This global spread in power across frequency band further quantifies the strength of a signal at a specific frequency and allows for information transfer across wider frequency range. The simulation, for a power spectrum *P* is described as follows:

(2) $$P=L+\overset{N}{\underset{n=0}{\sum }}G_{n}$$

Where *P* is the linear sum of the aperiodic signal *L* and *N* Gaussian peaks *Gn*. For each peak, Gaussian function $G_{n}$ is fitted which is modelled as the following:

(3) $$G_{n}=a*\exp^{\left(-\frac{{\left(F-C\right)}^{2}}{2w^{2}}\right)}$$

Where *a* denotes the amplitude, *c* denotes the CF, *w* denotes the BW of the Gaussian. *F* is the frequency vector. Subsequently, all fitted Gaussians were subtracted from the original power spectrum to get a peak-removed power spectrum (PRPS). Finally, a 1/f signal is estimated from this PRPS using Equation 4, representing the actual cortical noise. Exponential function in semilog-power space (logged power values and linear frequencies) is used to model the aperiodic signal (initial and final fit both), *L*, as:

(4) $$L=b-log(k+F^{\chi }).$$

Where *b* denotes the broadband offset, *χ* is the slope, and *k* is the knee parameter, which depends on the bend in the aperiodic signal. *F* is the frequency vector. The FOOOF model described in Equation 2 fits the power of a given sensor signal by estimating a linear function *L* for the aperiodic component of the signal and each oscillatory contribution *Gn* is modelled as Gaussian peaks. Moreover, estimated power was fitted across the entire frequency range of 1–45 Hz by as no knee was expected in the MEG recordings across the 1- to 50-Hz frequency range (Miller et al., 2009). The number of oscillatory components is determined from the data, with the option to set a maximum number of components as a parameter. The general model assumption here is that oscillatory and aperiodic processes are distinct and separable. Algorithm was implemented using custom python scripts on the python3 version.

**Figure 3.**
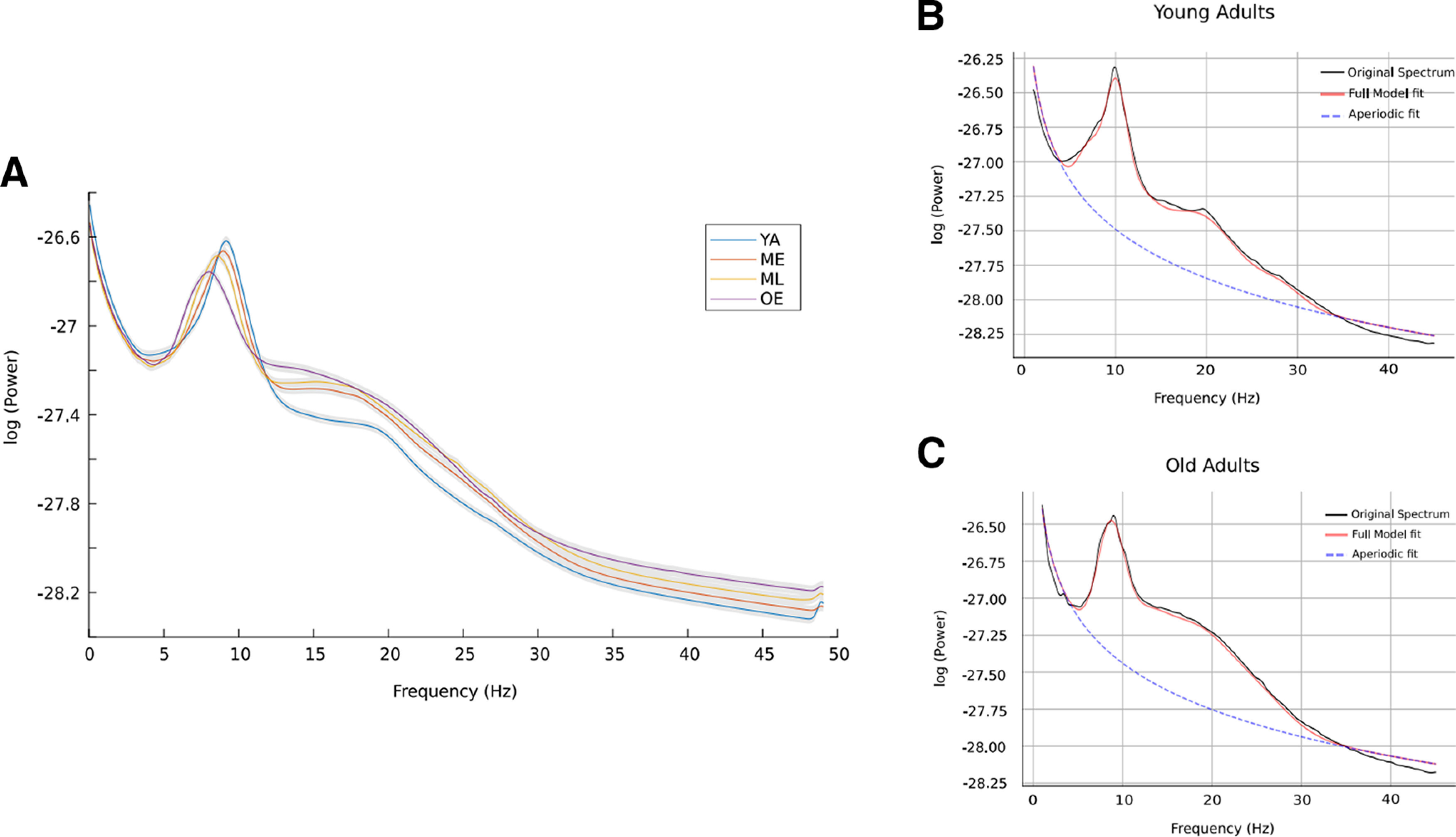
Parameterization using FOOOF model. (***A***) Power spectrum of all age-groups after removing the 1/f activity component. (***B***) (***C***) FOOOF model fit for young and old adults. Power spectrum without model fitting and for the other two age categories along with their statistics is shown in Extended Data Figure 3-1. Other extended analysis for different age groups is shown in Extended Data Figures 3-2, 3-3, 3-4, 3-5.

The model was fitted for individual subject and output parameters were averaged across subjects for each group (Fig. 3*A*). The settings for the algorithm were set as: (1) peak_width_limits = [0.5, 12]; (2) min_peak_height = 0; (3) max_n_peaks = 12; (4) peak_threshold = 2; (5) aperiodic_mode = “fixed”; and (6) verbos = “true.” Oscillations were *post hoc* grouped into θ (4–8 Hz), α (8–12 Hz), and β (13–30 Hz). For estimating the topographical dynamical changes, the brain was segmented into five non-overlapping regions: frontal (number of sensors = 26), parietal (number of sensors = 26), occipital (number of sensors = 24), and right and left temporal (number of sensors = 26).

10.1523/ENEURO.0224-21.2021.f3-1 Extended Data Figure 3-1 A, Power spectrum without model fitting. ***B***, ***C***, FOOOF-power spectrum model for ME and ML age groups, along with its statistics. Download Figure 3-1, EPS file.

10.1523/ENEURO.0224-21.2021.f3-2 Extended Data Figure 3-2 Power spectrum in log-log space. Download Figure 3-2, EPS file.

10.1523/ENEURO.0224-21.2021.f3-3 Extended Data Figure 3-3 ***A***, Aperiodic component of different age groups. ***B***, Boxplot of aperiodic features across different age groups derived from the component. Download Figure 3-3, EPS file.

**Figure 4.**
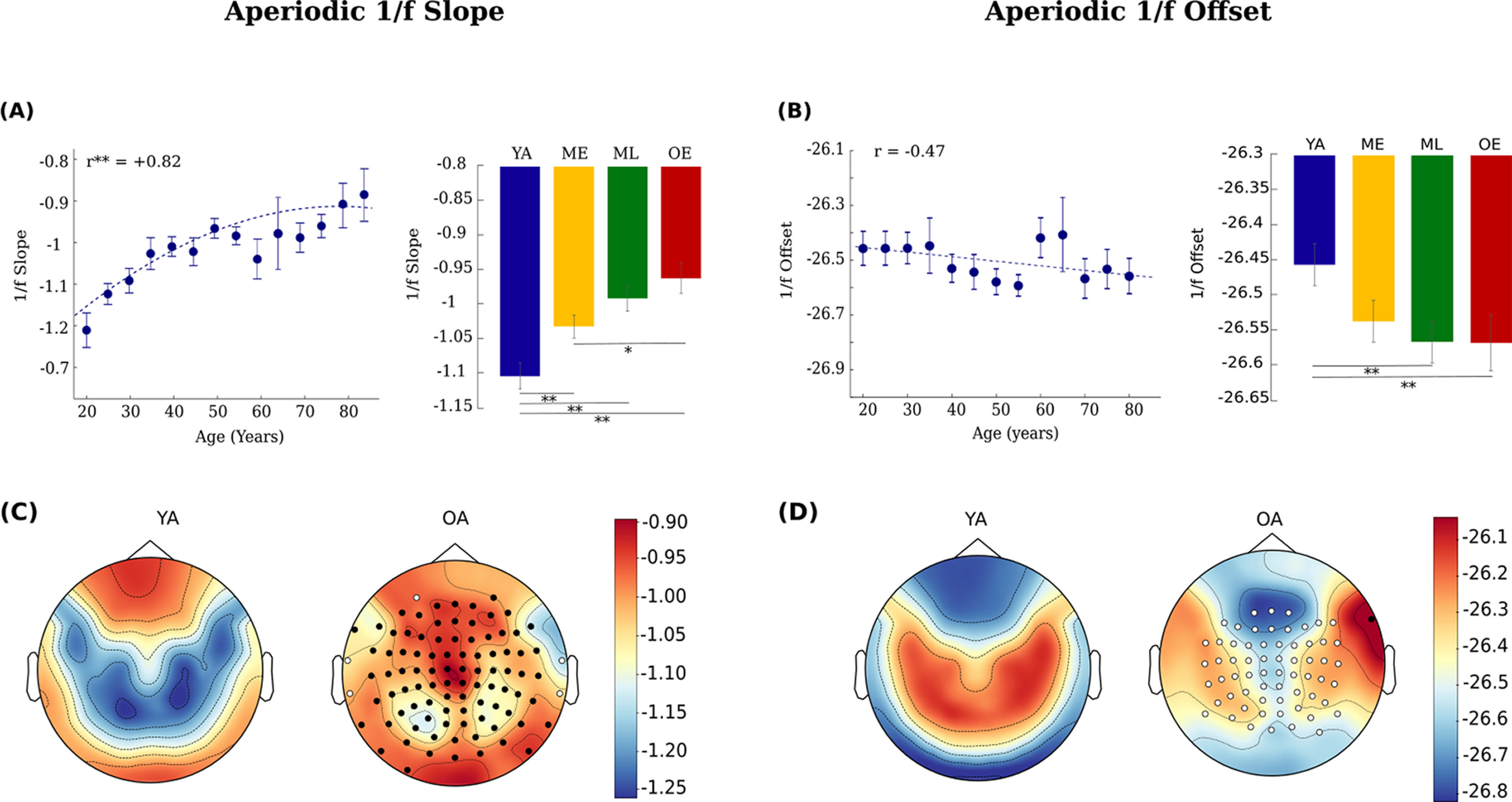
Aperiodic 1/f Slope and Offset. (***A***) left, 1/f slope as a function of age. Right, 1/f slope for four age groups. (***B***) left, 1/f offset as a function of age. Right, 1/f offset for four age groups. *r* represents the correlation value. The dashed line represents a linear regression fit. Error bar denotes SEM. (***C***) (***D***) Aperiodic 1/f slope and 1/f offset spatial topography for young (YA) and old (OA). Clusters of sensors with significant positive and negative differences in 1/f slope and 1/f offset between the OA and YA group are represented with black and white dots, respectively.

10.1523/ENEURO.0224-21.2021.f3-4 Extended Data Figure 3-4 Variability of aperiodic component across individual subjects in each age group. Download Figure 3-4, EPS file.

**Figure 5.**
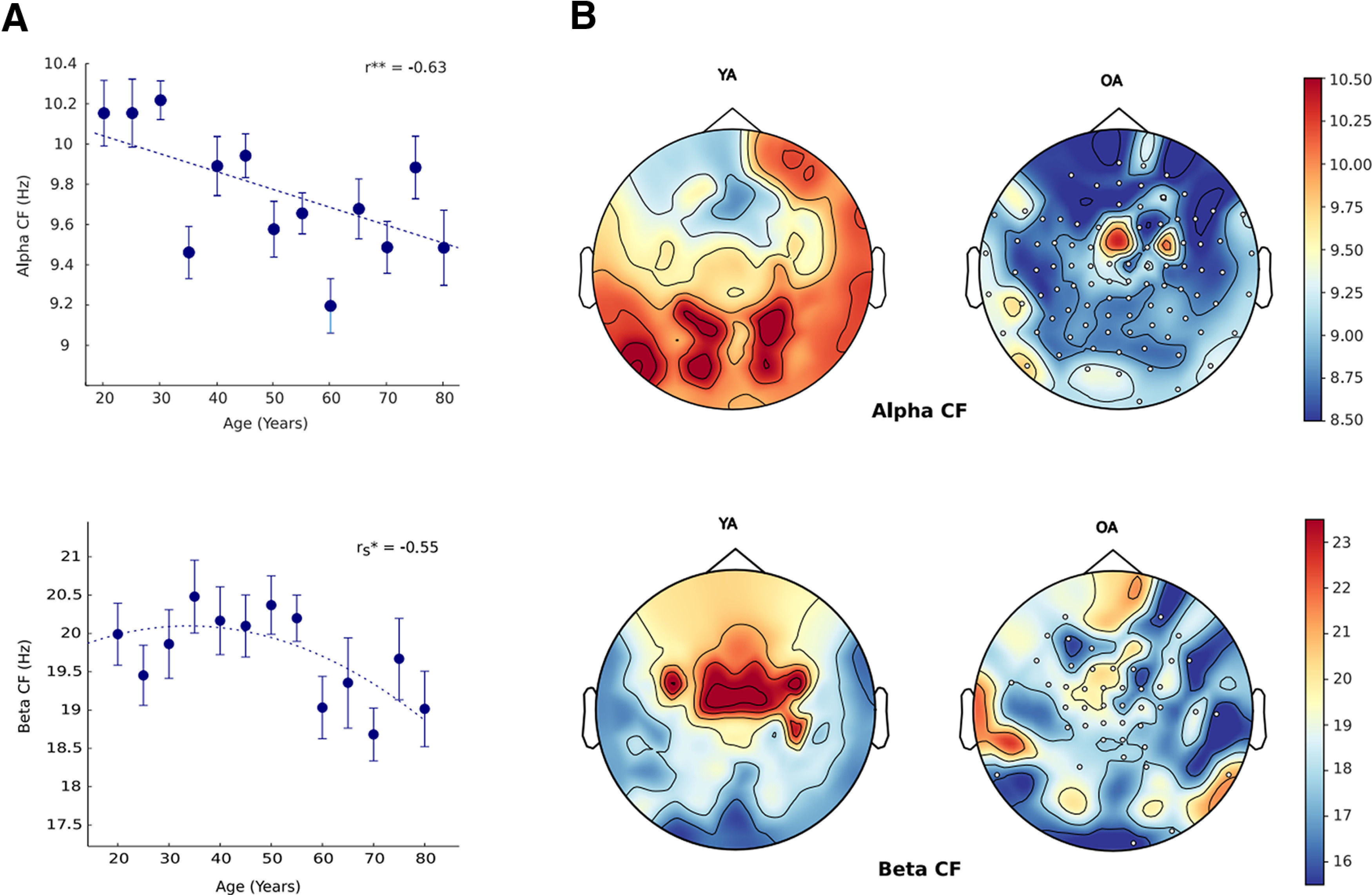
α And β PF as a function of age. (***A***) Top: PAF as a function of age. Bottom, β PF with age. *r* represents the correlation coefficient. The dashed line represents a linear regression fit. Error bar denotes SEM. (***B***) Top: Spatial topography for PAF and β PF for young (YA) and old (OA). Clusters of sensors with negative differences which contribute to the decrease are represented as white dots. Dominant frequency and power for YA and OA are shown in Extended Data Figure 5-1.

10.1523/ENEURO.0224-21.2021.f3-5 Extended Data Figure 3-5 Association between 1/f aperiodic slope with periodic components of the dominant oscillations. Download Figure 3-5, EPS file.

##### BR measures

Additionally, we estimated the BRs which reflect the quantitative measure of oscillatory activity and are investigated in different cognitive processes; however, they also get impacted by the 1/f background noise (Donoghue et al., 2020). After removing the aperiodic signal using a parametrization method proposed by the FOOOF toolbox, periodic values were estimated. Thus, after implementing appropriate parametrization of the aperiodic component of the signal power spectra BR values were re-estimated to indicate the true power changes and finally, were grouped into different frequency bands of interest. For each participant, we calculated the ratio of periodic components of different frequencies and averaged across participants for age bin-wise distribution. BR of all the periodic components for each frequency band was then calculated by dividing the average of low band periodic features by the average of high band periodic features. We calculated frequency-specific BRs of all periodic features.

(5) $$Band\;Ratio\left(X\right)=\frac{Avg\left(X_{LowBand}\right)}{Avg(X_{HighBand)}},$$

where *X* = *PW*, *CF*, *BW*.

#### Statistical analysis

We performed both categorical as well as continuous analyses to capture different aspects of age-associated functional differences. For the continuous analysis, we divided the total number of participants into bins of five years starting from 18 years, a total of 14 bins, and the center value was taken to be the representative age for each bin. For the categorical analysis, we divided data into the following age stratifications (18–35, 36–50, 51–64, and 66–88 years) to get insights about different important stages of adult lifespan and comparison with previous works (Chan et al., 2014; Sahoo et al., 2020). To clearly delineate the effects of periodic and aperiodic features on VSTM features, it was necessary to illustrate the effect in different load conditions. Only set size 2 and set size 4 are reported in the main manuscript, where we have categorized the set sizes in two load conditions: set size 2 as a low load condition and set size 4 as a high load condition. We have provided the findings for behavioral features of other set sizes as Extended Data Figs 9-2, 9-3, 9-4.

##### Correlation analysis

Depending on the data distribution, Pearson or Spearman’s correlation was used to estimate the strength between two variables. Estimated functional changes (PF, aperiodic, and BR measures) and VSTM task measures were correlated with age. Finally, VSTM task measures were correlated with the functional changes that occurs with aging to elucidate the role of these functional measures in determining behavioral responses.

##### Regression analysis

Linear and nonlinear regression were performed separately considering each power, CF, BW, slope, offset, and BRs of periodic features, as the estimated measures (R) of functional changes and precision, reaction time (RT), metacognitive awareness (d), and memory capacity (k), as the estimated measures (R) of the VSTM behavioral task, while keeping age as an explanatory variable:

(6) $$R=\beta_{0}+\beta_{1}*(age)$$

Linear regression was performed using *fitlm* MATLAB function. To capture the potential nonlinear effects of age, we also added second order polynomial terms to the model, such as:

(7) $$R=\beta_{0}+\beta_{1}*\left(age\right)*\beta_{2}*(age^{2})$$

Linear regression was also performed considering each VSTM task measure as response variable (*R*) and the functional measure as the explanatory variable (*E*).

(8) $$R=\beta_{0}+\beta_{1}*(E)$$

All regression tables are provided in the supplementary document (Extended Data Tables 8-1, 9-1, 10-1, 10-2, 11-1, 11-2, 11-3, 11-4). For estimating the significance, first normality of the data distribution was assessed using the Kolmogorov–Smirnov test. Based on the data distribution, parametric (*t* test) or nonparametric (Wilcoxon rank-sum test) was performed.

##### Code accessibility

The codes for all the analysis conducted in this paper is freely available to download from https://drive.google.com/drive/folders/1__74tFI1_VnHaV_-kJ46VAGMGiI04T2i?usp=sharing.

## Results

Using the FOOOF model, we fitted the PSD, and from the parametrization model fit, all the simulated Gaussian peaks were removed to analyze the background signal. Thereafter, the aperiodic component of the signal was fitted in the log-log space line (Extended Data Fig. 3-2), from which 1/f Slope and offset were extracted for each participant. Periodic features, CF, power (PW), BW, were estimated using peak parameters from the fitted model (see Materials and Methods). To check whether the parameterization using the simulated FOOOF model is able to capture lifespan associated changes, we first simulated the model for young and old adults. The model well captured the well-established lifespan associated with slowing down of PAF (Fig. 3). Original spectrum, aperiodic fit and full model are being depicted in Figure 3*B*,*C* for YA and OA group, respectively (extended analyses are shown in Extended Data Figs. 3-1, 3-2, 3-3, 3-4, 3-5 for model’s output parameters of ME and ML groups). To capture the dynamical changes in the dominant oscillations (highest power peak across all frequencies) across the adult lifespan, the CF, PW, and BW of the dominant oscillations were also extracted for young and old adults.

### Topographical distribution of aperiodic 1/f component of the signal with age

#### Increase in aperiodic 1/f slope and decrease in 1/f intercept

Aperiodic 1/f slope increases significantly when age was treated as a pseudo-continuous variable ( $\beta_{1}$= +0.0034901, *R*^2^ = 0.584, *p* = 0.003) whereas 1/f offset does not show significant decrease across the adult lifespan ( $\beta_{1}$ = −0.0033423, *R*^2^ = 0.3, *p* = 0.03; Table 3, Fig. 4*A*,*B*, Extended Data Fig. 3-3). Categorical analysis also confirmed significant difference in the 1/f slope between the OA versus YA (*t*_(140)_ = 4.38, *p* < 0.0001), ML versus YA (*t*_(140)_ = 4.07, *p* = 0.02), ME versus YA (*t*_(140)_ = 2.7749, *p* = 0.007), ME versus OA (*t*_(140)_ = −2.4581, *p* = 0.02). Categorical difference in 1/f offset was also found between OA versus YA (*t*_(140)_ = 2.0345, *p* = 0.0457) and ML versus YA (*t*_(140)_ = −2.3441, *p* = 0.02; Fig. 4*A*,*B*). Aperiodic fit and full model are being depicted in Figure 3 for YA and OA group, respectively, and within-group analysis revealed more variability in aperiodic features in the OAs (slope: SEM = 0.023; offset: SEM = 0.0404) compared with YA (slope: SEM = 0.014; offset: SEM = 0.0364; Extended Data Figs. 3-3, 3-4). Figure 4*C*,*D* shows variability in spatial topographies of aperiodic 1/f slope and offset for young and old.

**Table 3:** Effect of age on periodic and aperiodic features

Effect	Response variable	*F* value	Coefficient β_1_	*R* ^2^	*p* value
Age			Aperiodic features		
	1/f slope	26	+0.0034901	0.584	0.003262
	1/f offset	5.35	−0.0033423	0.308	0.0894
			Periodic features		
	Power				
	θ	6.82	+0.0050947	0.363	0.0227
	α	36.3	−0.0059263	0.751	0.0000599
	β	28.7	+0.002496	0.705	0.000172
	Central frequency				
	θ	0.577	+0.0029928	0.0459	0.462
	α	7.32	−0.010234	0.41	0.0205
	β	10.3	−0.024068	0.462	0.00751
	Bandwidth				
	θ	2.15	−0.0062634	0.15	0.168
	α	5.05	+0.001472	0.3	0.056
	β	17	+0.040345	0.58	0.00141

### Topographical distribution of PF with age

#### Age-associated slowing of central α frequency (CAF) and β frequency

For each participant, PF was quantified by estimating the peak power value within the 8–12 and 13–30 Hz for α and β range, respectively. Each participant’s PF was then averaged to get the group-wise estimation of CAF. Visual inspection revealed bin 65 to be the outlier (for CAF). After removing the outlier, significant linear age-related decline was found ( $\beta_{1}$ = −0.010234, *R*^2^ = 0.4, *p* = 0.02); however, central β frequency (CBF) showed nonlinear decrease with age ( $\beta_{1}$ = −0.024068, *R*^2^ = 0.462, *p* = 0.007; Fig. 5*A*). Categorical analysis also revealed significant CAF differences between YA versus OA (*t*_(140)_ = 4.7551 *p* = 0.00001), YA versus ME (*t*_(140)_ = 3.4198, *p* = 0.001), and YA versus ML (*t*_(140)_ = 4.8826, *p* = 0.000001), and for CBF between YA versus OA (*t*_(140)_ = 1.912, *p* = 0.03). Almost all sensors were found to be contributing to the decrease in CAF in OA whereas the decrease in CBF was mainly contributed by the central sensors (Fig. 5*B*). No significant difference was found in the frequencies of dominant oscillation; however, the power of the respective dominant frequencies was found to be significantly different between YA and OA (extended analyses are shown in Extended Data Fig. 5-1).

10.1523/ENEURO.0224-21.2021.f5-1 Extended Data Figure 5-1 Dominant frequency (***A***) and respective power (***B***) for YA and OA. Download Figure 5-1, EPS file.

#### Age-associated functional power change in α, θ, and β frequency

**Figure 6.**
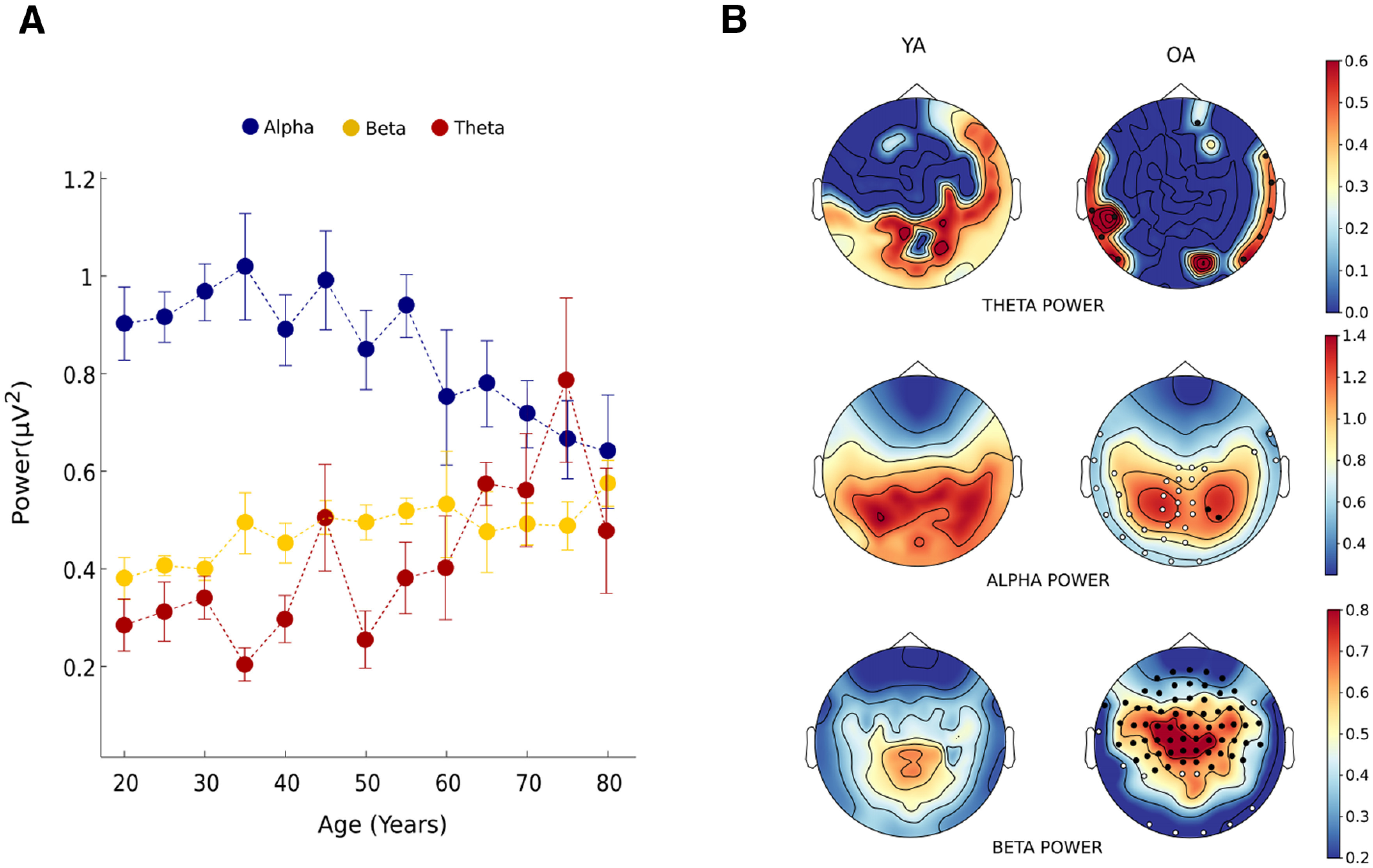
Parameterized global power as a function of age. (***A***) Increase in θ and β power whereas decrease in α power with age. Error bar represents SEM. (***B***) Spatial power topography of θ, α, and β for young (YA) and old adults (OA). Clusters of sensors with significant positive and negative differences between the OA and YA group are represented with black and white dots, respectively. Frequency specific power as a function of age across different sensors is shown as Extended Data Figure 6-1.

We found a robust decline of α power with age ( $\beta_{1}$ = −0.0059263, *R*^2^ = 0.75, *p* = 0.00005; Fig. 6*A*). Visual inspection suggests that sensor level α power difference was mainly contributed by the occipital, parietal and left temporal sensors (Fig. 6*B*). Significant difference was found between OA versus YA (*t*_(140)_ = −3.038, *p* = 0.003), OA versus ME (*t*_(140)_ = −2.2008, *p* = 0.03), and OA versus ML (*t*_(140)_ = −2.2252, *p* = 0.029). OAs showed higher θ power (M = 0.56 ± 0.04) than younger adults (M = 0.32 ± 0.02; *t*_(140)_ = 2.4733, *p* = 0.023). Significant age effect was also observed with increase in θ power ( $\beta_{1}$= 0.0050947, *R*^2^ = 0.363, *p* = 0.022; Fig. 6*A*), which was mainly contributed by the temporal sensors. In addition, aging was also associated with an increase in β power ( $\beta_{1}$= 0.002496, *R*^2^ = 0.70, *p* = 0.0001; Fig. 6*A*). Spatial topographies showed Central and frontal sensors to be contributing to this age-related increase in global β power (Fig. 6*B*). Categorical analysis revealed significant differences in β power between the YA versus OA (*t*_(140)_ = −4.3693, *p* = 0.00004), YA versus ME (*t*_(140)_ = −3.0103, *p* < 0.003), and YA versus ML (*t*_(140)_ = −4.4158, *p* = 0.00003). Extended analysis is shown in Extended Data Figure 6-1 displaying the sensor-wise distribution of frequency-specific power as a function of age.

10.1523/ENEURO.0224-21.2021.f6-1 Extended Data Figure 6-1 Frequency specific power as a function of age across different sensors. For Frontal sensors, θ power is not shown as only few age groups showed peaks in θ range. Error bar represents SEM. Download Figure 6-1, EPS file.

#### Increase in β BW with age

**Figure 7.**
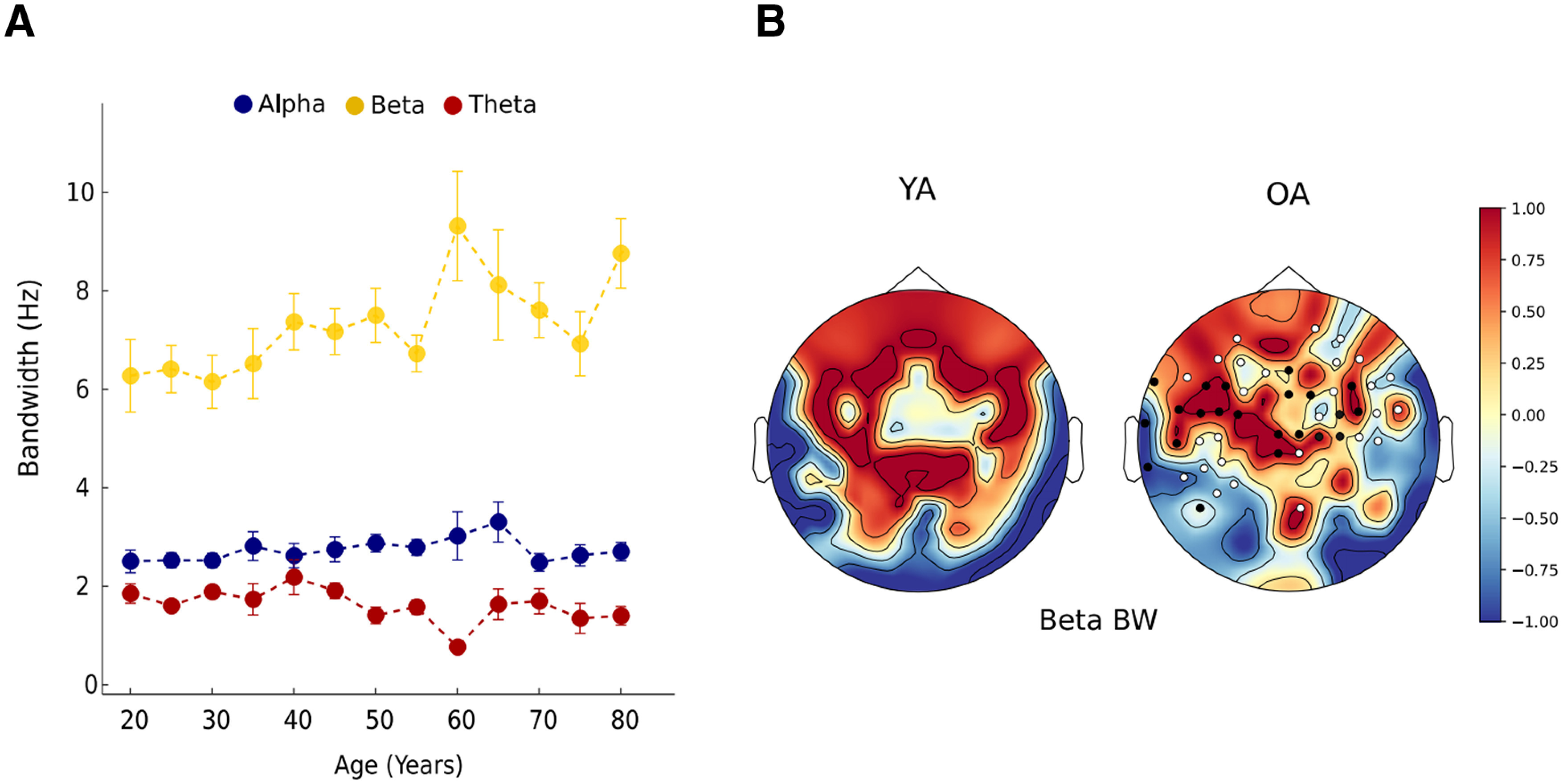
Global frequency-specific BW with age. (***A***) Bar graph for each age group, representing BW for each frequency band. (***B***) Spatial topography of β BW for young (YA) and old adults (OA). Clusters of sensors with significant positive and negative differences between the OA and YA group are represented with black and white dots, respectively. All the extended analyses are shown as Extended Data Figures 7-1, 7-2.

BW reflects the spread of power in the respective frequency range, which for the β band was found to be increased across the adult lifespan ( $\beta_{1}$ = 0.040345, *R*^2^ = 0.58 *p* = 0.001; Fig. 7*A*). Significant group-wise difference was also seen between YA versus OA (*t*_(140)_ = −3.1586, *p* = 0.0024), YA versus ME (*t*_(140)_ = −1.9843, *p* = 0.049). Increase in β BW indicates that the β power tends to be more distributed within the frequency band as we age. This increase was mainly observed over left temporal and central sensors (Fig. 7*B*). BW for α and θ frequency band did not differ across age groups (Extended Data Fig. 7-1). For sensor topography refer to Extended Data Figure 7-2.

10.1523/ENEURO.0224-21.2021.f7-1 Extended Data Figure 7-1 Frequency specific BW for different age groups. Download Figure 7-1, EPS file.

10.1523/ENEURO.0224-21.2021.f7-2 Extended Data Figure 7-2 Spatial topography of θ BW (***A***) and α BW (***B***) for YA and OA. Download Figure 7-2, EPS file.

### Topographical Distribution of BRs with age

BR measures have been argued to be a marker of various cognitive measures in healthy adults as well as in pathologic conditions (Schutter and Van Honk 2005; Kamiński et al., 2011; Trammell et al., 2017) which also gets affected by 1/f activity. We investigated how these global BRs change with age after effectively removing the background 1/f activity. We looked at θ/α, θ/β, and α/β BRs, where the ratio of all periodic features (PW, CF, BW) was analyzed for each frequency band. For all BR measures, we calculated correlations between the spectral features of each oscillation-band and age. Here, we showed the global change (averaged across all sensors) in the BR measures across the lifespan.

**Figure 8.**
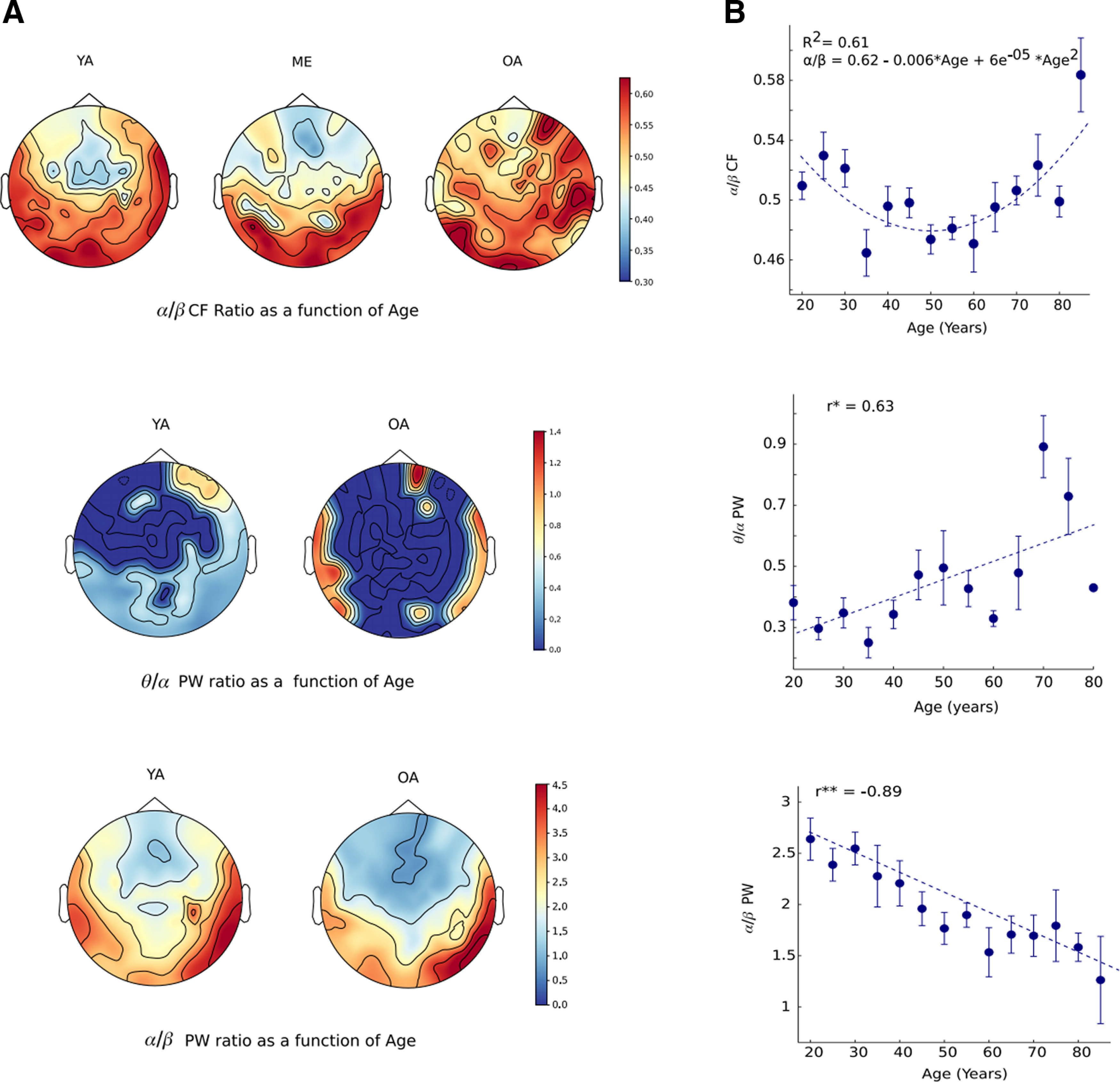
Spatial topography of BR measures as a function of age. (***A***), Spatial topography of α/β PF ratio ( α/βCF; top), α/β power ratio (α/β PW; center), and θ/α power ratio (bottom) for young (YA) and old adults (OA). (***B***), Regression fit model for each of the ratio measures keeping age as an explanatory variable. Error bar represents SEM. *R*^2^ represents goodness of fit, and *r* represents the correlation coefficient. All the extended analyses are shown as Extended Data Figures 8-1, 8-2, 8-3, 8-4, 8-5. A detailed regression table is provided in the Extended Data Table 8-1.

The age-associated nonlinear change was mostly observed in frontal and parietal sensors (Fig. 8*A*). For age categories, we found a significant difference between OA versus ME (*t*_(134)_ = 2.38, *p* = 0.018), OA versus ML (*t*_(134)_ = 3.19, *p* = 0.0018), YA versus ME (*t*_(138)_ = 3.30, *p* = 0.0012), and YA versus ML (*t*_(138)_ = 4.09, *p* = 0.00007). For the CF ratio, we found α/β ratio to vary nonlinearly (quadratic) with age (β_1_ = −0.0059138, *R*^2^ = 0.61 *p* = 0.005), whereby first decreases for middle age and subsequently an increase for older age participants suggesting an overall U-shaped response of α/β ratio through lifespan (Fig. 8*B*). No significant difference was found between the categorical age groups for θ/α and θ/β peak ratios (Extended Data Figs. 8-1, 8-2; Extended Data Table 8-1).

10.1523/ENEURO.0224-21.2021.f8-1 Extended Data Figure 8-1 θ/α PF ratio (***A***) and θ/β PF ratio (***B***) for YA and OA. Download Figure 8-1, EPS file.

10.1523/ENEURO.0224-21.2021.f8-2 Extended Data Figure 8-2 θ/β power ratio for YA and OA. Download Figure 8-2, EPS file.

10.1523/ENEURO.0224-21.2021.f8-3 Extended Data Figure 8-3 ***A***, θ/β BW. ***B***, θ/α BW as a function of age. Download Figure 8-3, EPS file.

10.1523/ENEURO.0224-21.2021.f8-4 Extended Data Figure 8-4 ***A***, Topoplots of θ/β BW. ***B***, θ/α BW for YA and OA. Download Figure 8-4, EPS file.

10.1523/ENEURO.0224-21.2021.f8-5 Extended Data Figure 8-5 Association between 1/f slope with α/β power ratio. Download Figure 8-5, EPS file.

10.1523/ENEURO.0224-21.2021.tab8-1 Extended Data Table 8-1 Regression table for global frequency BRs with age. *F* value, β coefficient, goodness of fit, and significance of the model are reported. Download Table 8-1, DOC file.

Power Ratio of θ/α was found to be positively correlated with age (β_1_ = 0.0057613, *R*^2^ = 0.40, *p* = 0.02) whereas α/β power ratio was negatively correlated with age (β_1_ = −0.018116, *R*^2^ = 0.85, *p* = 0.000001; Fig. 8*B*). Significant categorical difference was found for θ/α power ratio between YA versus OA (*t*_(136)_ = 4.9615, *p* = 0.000002), YA versus ME (*t*_(138)_ = 2.75, *p* = 0.0067), YA versus ML (*t*_(138)_ = 4.92, *p* = 0.000002), ME versus OA (*t*_(134)_ = 2.24, *p* = 0.02). No significant correlation was found for θ/β power ratios with age (*R*^2^ = 0.2, *p* = 0.1). For α/β power ratio, significant categorical difference was found between YA versus OA (*t*_(76)_ = −4.6, *p* = 0.00001), ME versus OA (*t*_(59)_ = −3.33, *p* = 0.0015), and ML versus OA (*t*_(62)_ = −2.46, *p* = 0.01). No significant difference was found between the categorical age groups for θ/β power ratio.

BW ratio of θ/β and θ/α was found to be negatively correlated with age (extended analysis is shown in Extended Data Figs. 8-3, 8-4). Categorical analysis revealed differences between the YA versus OA (*t*_(80)_ = 2.21, *p* = 0.029) for θ/β BW ratio. No significant difference was found between the categorical age group for α/β BW ratio.

After characterizing the normative pattern of true oscillatory changes across age, we tested our hypothesis by carrying out regression analysis whereby keeping 1/f noise, periodic features as an explanatory variable and behavioral measures as response variable (see Materials and Methods). All correlations were performed after regressing out the age.

We first analyzed the behavioral responses of the same participants in the VSTM task to replicate the well-established cognitive decline with age. Grouping of participants in the age groups and bins were done similarly.

### Behavioral results: age-related cognitive decline reflected in performance

#### Precision

**Figure 9.**
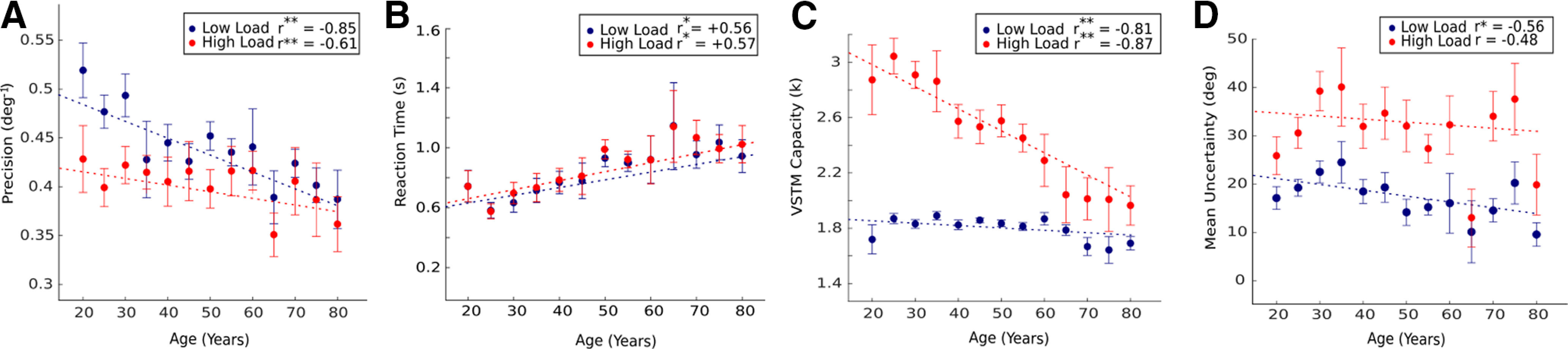
Effect of memory load and age on VSTM. VSTM measures (***A***) precision, (***B***) RT, (***C***) VSTM capacity (k), and (***D***) mean uncertainty as a function of age. Low load and high load indicate set sizes 2 and 4, respectively. The dashed line represents the linear regression fit. Error bar represents the SEM for each age bin. Asterisks indicate significance. All the extended analyses are shown as Extended Data Figures 9-1, 9-2, 9-3, 9-4 and Extended Data Table 9-1.

As expected, precision becomes worse with memory load and age. Overall precision was high for the set size 1 (61.1% SEM 2%) as compared with set sizes 2 (48.7% SEM 1.9%), 3 (39.6% SEM 1%), and 4 (39% SEM 0.7%). Continuous analysis revealed significant decrease in precision with age in both low and high load conditions (low load, *r* = −0.85, *p* < 0.01, high load, *r* = −0.61, *p* < 0.05; Fig. 9*A*; extended analysis is shown in Extended Data Fig. 9-1). Categorical analysis between the groups revealed significant differences in the mean of YA versus OA (YA = 0.48 ± 0.008, OA = 0.30 ± 0.005, *p* < 0.0001), YA versus ME (YA = 0.48 ± 0.008, ME = 0.45 ± 0.007, *p* < 0.001), YA versus ML (YA = 0.48 ± 0.008, ML = 0.43 ± 0.007, *p* < 0.0001), and ME versus OA (ME = 0.45 ± 0.007, OA = 0.30 ± 0.005, *p* < 0.0001) groups. Within-group analysis also revealed significant increase in precision with increase in memory load (Extended Data Fig. 9-2; Extended Data Table 9-1).

10.1523/ENEURO.0224-21.2021.f9-1 Extended Data Figure 9-1 VSTM measures as a function of age. Participants with same age were grouped together (dots), size of the dot represents the SEM of the group. Shaded area is the 95% confidence interval. Download Figure 9-1, EPS file.

10.1523/ENEURO.0224-21.2021.f9-2 Extended Data Figure 9-2 Precision across age groups and set size. Download Figure 9-2, EPS file.

10.1523/ENEURO.0224-21.2021.f9-3 Extended Data Figure 9-3 Uncertainty across age groups and set size. Download Figure 9-3, EPS file.

10.1523/ENEURO.0224-21.2021.f9-4 Extended Data Figure 9-4 VSTM capacity across age groups for different set sizes. Download Figure 9-4, EPS file.

10.1523/ENEURO.0224-21.2021.tab9-1 Extended Data Table 9-1 Regression table for VSTM measures with age. *F* value, β coefficient, goodness of fit, and significance of the model are reported. Download Table 9-1, DOC file.

#### Reaction Time

Overall RT was higher for the set size 4 (910.2 ± 21.6 ms) as compared with set size 1 (878.7 ± 19.9 ms), 2 (870.4 ± 19.9 ms), and 3 (882.6 ± 21.4 ms) but increases significantly with age [low load (*r*) = +0.57, *p* < 0.05, high load (*r*) = +0.56, *p* < 0.05; Fig. 9*B*]. Group analysis also revealed significant difference between YA versus OA (YA = 668 ± 35.4, OA = 1009 ± 38.9, *p* < 0.00001), YA versus ME (YA = 668 ± 35.4, ME = 828.8 ± 41.5, *p* = 0.002), YA versus ML (YA = 668 ± 35.4, ML = 886 ± 33.4, *p* < 0.0001), ME versus ML (ME = 828.8 ± 41.5, ML = 886 ± 33.4, *p* = 0.03), ME versus OA (ME = 828.8 ± 41.5, OA = 1009 ± 38.9, *p* < 0.001), ML versus OA (ML = 886 ± 33.4, OA = 1009 ± 38.9, *p* = 0.05).

#### VSTM capacity (k)

VSTM capacity was found to decrease with age [low load (*r*) = −0.81 *p* < 0.001, high load (*r*) = −0.87, *p* < 0.001; Fig. 9*C*]. Categorical analysis between the group revealed significant difference between YA versus OA (YA = 1.84 ± 0.01, OA = 1.66 ± 0.02, *p* < 0.0001), YA versus ML (YA = 1.84 ± 0.01, ML = 1.79 ± 0.01, *p* = 0.004), ME versus OA (ME = 1.83 ± 0.01, OA = 1.66 ± 0.02, *p* < 0.0001), and ML versus OA (ML = 1.79 ± 0.01, OA = 1.66 ± 0.02, *p* < 0.001; results for all the set sizes are reported in Extended Data Fig. 9-4).

#### Mean uncertainty (metacognitive measure)

Subjective uncertainty was higher in set 4 (29.7 ± 1) as compared with set size 1 (11.8 ± 0.39), 2 (15.6 ± 0.5), and 3 (20.7 ± 0.67). After performing regression and correlation analysis, we found that subjective uncertainty significantly decreases with age in low load condition [low load (*r*) = −0.56, *p* < 0.05; Fig. 9*D*]. Suggesting that OAs tend to be more confident about their erroneous answers when the load is less. Categorical analysis revealed significant differences in the mean of YA versus OA (YA = 18.5 ± 0.97, OA = 14 ± 1, *p* < 0.001), YA versus ME (YA = 18.5 ± 0.97, ME = 16.07 ± 0.95, *p* = 0.02), YA versus ML (YA = 18.5 ± 0.97, ML = 14.85 ± 1.05, *p* < 0.001), and ME versus OA (ME = 16.07 ± 0.95, OA = 14 ± 1, *p* = 0.003). Within-group analysis also revealed significant increase in subjective uncertainty with increase in memory load (Extended Data Fig. 9-3)

### Aperiodic 1/f slope: predictive of all measures of VSTM

**Figure 10.**
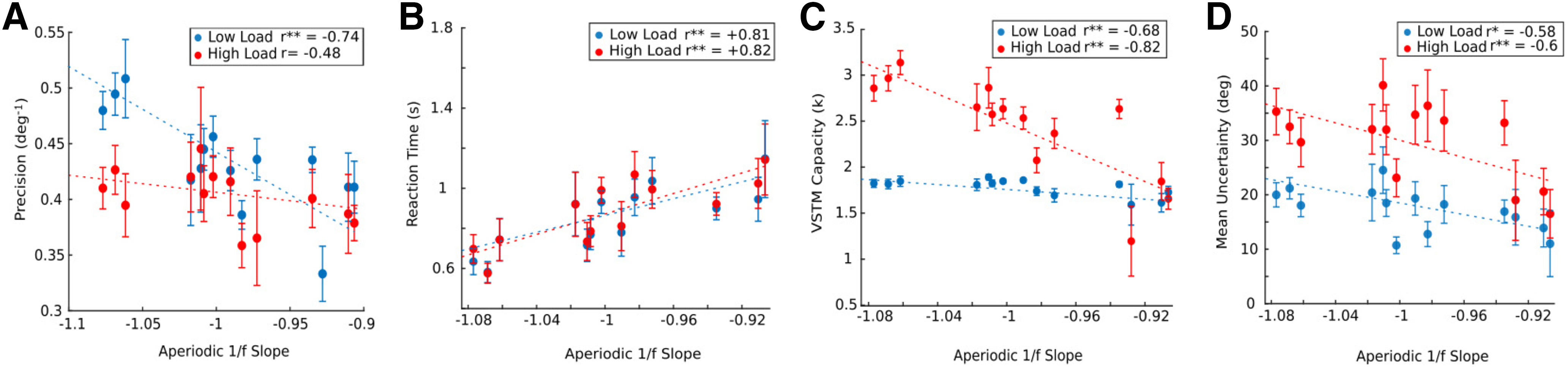
Aperiodic 1/f slope mediating VSTM performance. Linear regression model for VSTM measure (***A***) precision, (***B***) RT, (***C***) VSTM capacity (k), and (***D***) mean uncertainty as a response variable and aperiodic 1/f slope as an explanatory variable, after regressing out the age effect. The dashed line represents linear regression fir. Error bar represents SEM. Low load and high load indicate set sizes 2 and 4, respectively. *r* is Pearson’s coefficient. All the extended analyses are shown as Extended Data Table 10-1, Table 10-2.

We then assessed whether the VSTM performance was impacted by 1/f slope. As hypothesized, RS aperiodic 1/f noise was found to be predictive of decreased precision (low load: *r* = −0.74, *p* = 0.002, high load: *r* = −0.48, *p* = 0.08), memory capacity (low load: *r* = −0.68, *p* = 0.0007, high load: *r* =+0.82, *p* = 0.0003), mean uncertainty (low load: *r* = −0.58, *p* = 0.03, high load: *r* = −0.6, *p* = 0.02) and increased RT (low load: *r* = +0.56, *p* = 0.00005, high load: *r* = +0.57, *p* = 0.00005) in VSTM task (Fig. 10). However, we did not find any correlation between 1/f offset and behavioral measures (Extended Data Tables 10-1, 10-2). As aperiodic 1/f noise mediated a global effect on the VSTM performance, we further wanted to investigate how different oscillatory components mediate changes in the specific behavior measures in VSTM performance through lifespan.

10.1523/ENEURO.0224-21.2021.tab10-1 Extended Data Table 10-1 Regression table for VSTM measures with aperiodic slope. *F* value, β coefficient, goodness of fit, and significance of the model are reported. Download Table 10-1, DOC file.

10.1523/ENEURO.0224-21.2021.tab10-2 Extended Data Table 10-2 Regression table for VSTM measures with aperiodic 1/f offset. *F* value, β coefficient, goodness of fit, and significance of the model are reported. Download Table 10-2, DOC file.

### Precision increases with increase in α power and α/β power ratio

**Figure 11.**
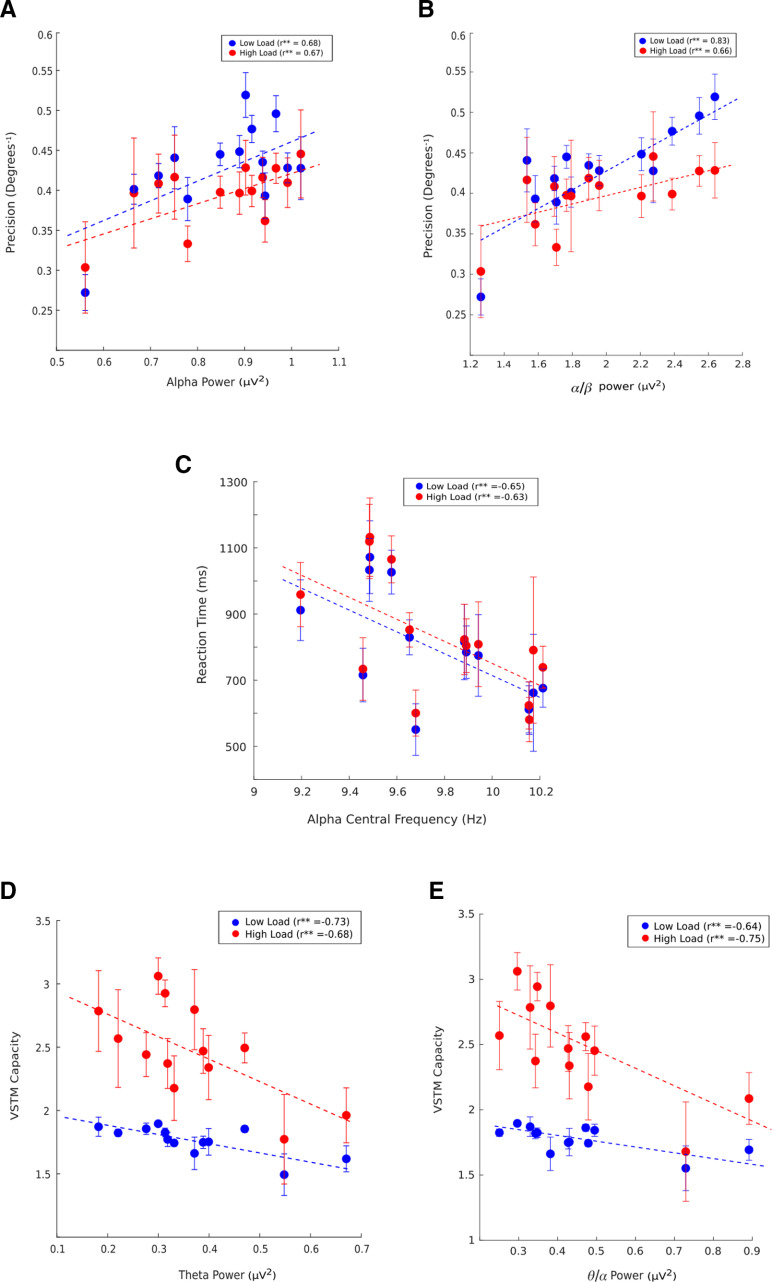
VSTM measures predicted by different oscillatory features and 1/f offset. (***A***) (***B***) Precision predicted by global α power and α/βpower ratio. (***C***) Speed of processing (RT) well predicted by global α speed (PAF). (***D***) (***E***) VSTM capacity predicted by global θ power and θ/αpower ratio. Age is regressed out. Low load and high load indicate set sizes 2 and 4, respectively. The dashed line represents the regression line. Error bar represents SEM, *r* corresponds to Pearson’s coefficient. All the extended analyses are shown as Extended Data Tables 11-1, 11-2, 11-3, 11-4.

Precision was found to be positively correlated with the α power for both low ( $\beta_{1}$ = 0.28 077, *R*^2^ = 0.425, *p* = 0.0115) and high ( $\beta_{1}$ = 0.17 617, *R*^2^ = 0.38, *p* = 0.0186) load condition (Fig. 11*A*). α/β

10.1523/ENEURO.0224-21.2021.tab11-1 Extended Data Table 11-1 Regression table for specific oscillatory features with VSTM measures. *F* value, β coefficient, goodness of fit, and significance of the model are reported. Download Table 11-1, DOC file.

10.1523/ENEURO.0224-21.2021.tab11-2 Extended Data Table 11-2 Regression table for α/β CF with VSTM measures. *F* value, β coefficient, goodness of fit, and significance of the model are reported. Download Table 11-2, DOC file.

10.1523/ENEURO.0224-21.2021.tab11-3 Extended Data Table 11-3 Regression table for α/β Power ratio with VSTM measures. *F* value, β coefficient, goodness of fit, and significance of the model are reported. Download Table 11-3, DOC file.

10.1523/ENEURO.0224-21.2021.tab11-4 Extended Data Table 11-4 Regression table for θ/α power ratio with VSTM measures. *F* value, β coefficient, goodness of fit, and significance of the model are reported. Download Table 11-4, DOC file.

Power ratio was also found to be a significant predictor of precision in low ( $\beta_{1}$ = 0.11 906, *R*^2^ = 0.69, *p* = 0.0002) and high load ( $\beta_{1}$ = 0.063459, *R*^2^ = 0.4, *p* = 0.008) conditions across lifespan (Fig. 11*B*). All the regression analysis results are shown for specific oscillatory features with VSTM measures in Extended Data Table 11-1.

### Speed of processing predicted by α speed

Speed of α is often related to the speed of processing which is generally measured as RT. As we observed that speed of α decreases and RT increases with age, we wanted to investigate whether this decrease in α speed affected the speed of processing in OAs. α Speed significantly predicted the speed of processing for both low ( $\beta_{1}$ = −340.82, *R*^2^ = 0.43, *p* = 0.0108) and high ( $\beta_{1}$ = −352.41, *R*^2^ = 0.39, *p* = 0.0158) load conditions (Fig. 11*C*).

### VSTM capacity predicted by θ power and θ/α band power ratio

We found a significant negative correlation of VSTM capacity with θ power (low load: *r* = −0.729, *p* = 0.004, high load: *r* = −0.679, *p* = 0.01) and θ/α power ratio (low load: *r* = −0.64, *p* = 0.01, high load: *r* = −0.75, *p* = 0.001), suggesting that these two play an important role in storing items in working memory. Regression analysis also revealed a significant role of θ power and θ/α power ratio in predicting VSTM capacity in low (θ power: $\beta_{1}$= −0.63 163, *R*^2^ = 0.53, *p* = 0.0046, θ/α: $\beta_{1}$= −1.5827, *R*^2^ = 0.569, *p* = 0.002) and high load conditions (θ power: $\beta_{1}$= −1.8862, *R*^2^ = 0.46, *p* = 0.011, θ/α: $\beta_{1}$= −0.35 435, *R*^2^ = 0.42, *p* = 0.01; Fig. 11*D*,*E*; Extended Data Tables 11-2, 11-3, 11-4).

## Discussion

Using three different measures (aperiodic 1/f slope and offset, PF, and BR of power in various frequencies), we have systematically investigated the spontaneous temporal dynamics and dynamical changes during resting state associated with healthy adult lifespan. Subsequently, we have demonstrated how these measures potentially link distinct behavioral responses during short-term working memory processing. Many previous studies in aging literature have demonstrated that task-relevant oscillatory changes accurately demarcate task performance in various cognitive domains (Clark, 2004; Cummins and Finnigan, 2007; Tóth et al., 2014; Proskovec et al., 2016; Rondina et al., 2019). As the resting-state serves as a baseline/control for the diverse task-related changes, it is crucial to characterize how specifically aging alters the normative brain network dynamics to impact cognition.

### The domain general effect of age-associated aperiodic 1/f activity

To track systematically healthy aging associated changes in neuronal oscillations through lifespan, a substantial number of previous studies have used narrowband power analysis that presumes that spectral power implies oscillatory power, without precisely separating the 1/f activity which in itself is dynamic and it impacts the oscillatory power which can lead to misinterpretation of the results. Extended analysis in Extended Data Figure 3-5 shows the relation between 1/f slope and dominant periodic features, indicating the interdependence of these two components and the necessity to detangle these. We approached this problem by applying a parameterization model (Donoghue et al., 2020), which detangles the periodic and aperiodic 1/f component.

**Figure 12.**
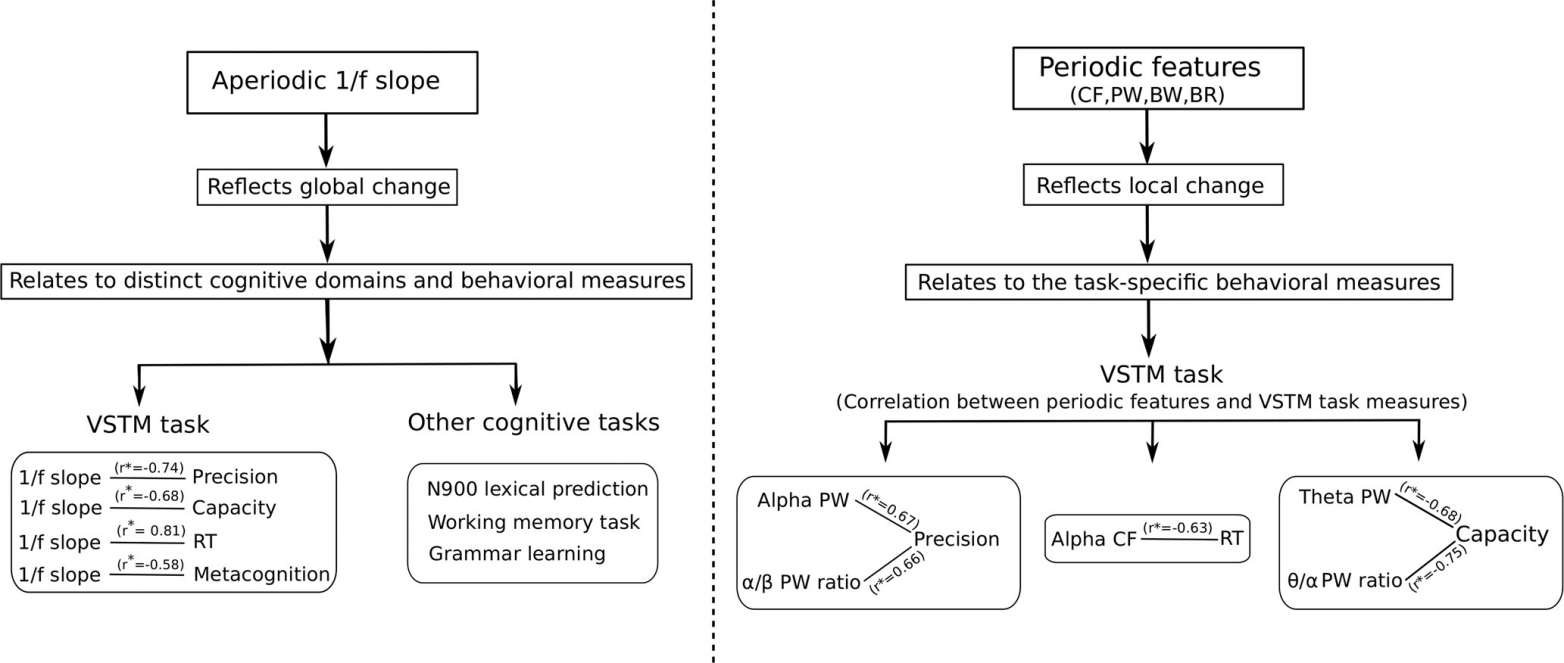
Left: Aperiodic 1/f slope index behavior in distinct cognitive domain: aperiodic 1/f slope increases globally which is reflected in performance in different cognitive tasks which includes VSTM task precision, cognitive capacity, RT, and metacognition and in other cognitive tasks (N900 lexical prediction, working memory, grammar learning) reported previously. *r* represents the Pearson’s correlation coefficient. Right: Periodic features index task-specific behavioral measures: periodic features (CF, BW, PW, BR) are more task-specific in nature. After regressing out the age, α band power, α/β BR index for precision; θ/α BR, θ power index for cognitive capacity; α CF index for RT. *r* represents the Pearson’s correlation coefficient.

Recent studies have considered 1/f slope as an index of noise in the brain (Ouyang et al., 2020; Donoghue et al., 2020). Ageing is associated with an increase in cortical neural noise, where studies have previously used RT as a proxy for the neural noise (Welford, 1981; Salthouse and Lichty, 1985; Cremer and Zeef, 1987). We observed 1/f slope of the MEG spectral power increases with age (flattening of PSD), which is suggestive of increased desynchronized neuronal activity (Hong and Rebec, 2012; Podvalny et al., 2015). Voytek and colleagues had suggested that this flattening of PSD slopes which they indexed for noise might be a hallmark of age-related cognitive decline (Voytek et al., 2015). Also, aperiodic 1/f noise is found to be very dynamic in nature and it has been shown to be predictive of performance in working memory tasks (Voytek et al., 2015), N400 lexical prediction (Dave et al., 2017), and in grammar learning (Cross et al., 2020). Our results depict the association between global change in the 1/f slope with capacity, speed, precision, and metacognition in short-term working memory processing. As 1/f activity is associated with several distinct domains of cognition, we further suggest that age-related increase in aperiodic 1/f slope does not necessarily mediate any domain-specific processing rather it affects domain general processing (Fig. 12).

Additionally, the increase in 1/f slope follows a monotonic nonlinear relationship with age suggesting that the rate of change in desynchronized neural activity is not necessarily constant across adult lifespan. We observe some deviation from the normal trend for both 1/f slope and offset in age-group 60–80, which might be because of the observed increased variance in the older group. Besides, aperiodic 1/f features were found to not only vary across subjects (more for elderly) but also across different sensors indicating substantial variability and idiosyncrasy. Although 1/f slope shows spatial heterogeneity in the young group, such as being less negative in the anterior sensors compared with the posterior sensors, older participants display a more homogenous distribution of less negative 1/f slope values. The broadband offset shows no significant deviation with age, but significant between-group differences were observed.

### Aperiodic 1/f activity, self-organized criticality, and nonlinear relationship with adult lifespan

At a more fundamental level, 1/f scale reflects the self-similar temporal properties of the self-organized critical states. Although the aim of this study is not to resolve this debate, however, we argue that 1/f activity could arise from potentially number of factors, e.g., altered tissue properties or self-organized criticality and transient stability with aging and change in underlying excitation-inhibition (E/I) balance (Bédard et al., 2006; Voytek et al., 2015; Gao et al., 2017; Naik et al., 2017; Naskar et al., 2021).

Criticality hypothesis, which proposes that the brain operates in a critical state, and alteration in criticality could be symptomatic or causative for certain pathologies (Hesse and Gross, 2014) seems to be operative here in terms reorganization of brain dynamics based on 1/f slope and offset and their relationship with cognitive performance. Literature suggests that neural networks at criticality exhibit properties for optimal performance such as information transmission and storage, metastable state, dynamic range and computational power (Beggs, 2008; Shew and Plenz, 2013). However, 1/f scale cannot alone be explained by criticality; rather emergence may lie in the local tissue properties (Bédard et al., 2006). Healthy aging is consonant with alteration of physiological properties in brain tissue (Aalami et al., 2003; Peters, 2006), therefore, it seems plausible to say that change in 1/f scale and increase in 1/f slope exhibiting nonlinear relationship with aging has its origin in the local tissue properties.

Subsequently, the association of 1/f slope with cognitive and metacognitive aspects of VSTM demonstrate the domain general effects of local tissue properties affecting self-organized criticality of functional brain states which in turn affects behavior.

### PF, BR relates to distinct aspects of memory processing

In oscillatory dynamics, we observe a significant decline in PAF with age as shown by previous studies (Voytek et al., 2015; Sahoo et al., 2020). This decrease in PAF was not found to be localized to specific sensors, rather a global significant decrease was observed (Fig. 5*B*). The speed of α is often associated with the speed of information processing therefore, higher α speed is needed for optimal performance in cognitive tasks (Surwillo et al., 1961) and determine the temporal resolution of visual perceptual integration (Samaha and Postle, 2015). Figure 11*C* shows that the RT of the participants or speed of memory retrieval is well predicted by global α speed. Hence, higher the speed of α, fast is the retrieval, and consequently lesser RT for younger adults. As aging is characterized by attentional difficulties in particular, a reduced capability to inhibit irrelevant information (McNab et al., 2015), therefore, α band power may have an important role in determining how accurately older individual’s recall the memorized items. However, a study by Vaden et al. (2012) demonstrated that older people do not use α power suppression to inhibit distractor’s information. In this work, we found that α power decreases with age, particularly over occipito-parietal sensors (Fig. 6), which significantly predicts the precision (Fig. 11*A*), along with α/β power which decreases with age (Griffiths et al., 2019; Fig. 8*B*). It plays a crucial role in suppressing irrelevant information, therefore, not being able to ignore distractions might be one of the reasons for low VSTM capacity found in OAs.

The relevance of θ CF in determining VSTM capacity in a task is well known in the literature. A study by Moran et al. (2010) shows that both slow and fast θ frequencies correlated to the high memory capacity, distributed across different networks. In the context of aging, we observe that θ CF slightly increases for older subjects as compared with younger adults which may itself affect the storing capacity. There are studies which have observed an increase in RS θ power in OAs (for review, see Klimesch, 1999; Klass and Brenner, 1995) others have reported θ power decrease in resting as well as in the task with age (Babiloni et al., 2006; Cummins and Finnigan, 2007; Leirer et al., 2011; Vlahou et al., 2014). However, we found an increase in θ power with age, which significantly predicted the VSTM capacity (Figs. 6*A*, 11*E*) along with θ/α power ratio (TAR), which also significantly increases with age (Fig. 8). Few studies including the study by Trammell et al. (2017) found decreased performance in RM correlated with increased TAR in old adults. We found substantial variability in the presence of θ power in participants. For instance, in young groups, the θ was not observed over frontal and left temporal sensors, whereas in older participants the θ power was observed only over temporal sensors. Additionally, we also observed an increase in β power with age which is well reported in the literature, generally associated with the movement-related activity (Ishii et al., 2017; Sahoo et al., 2020) but we also observed a significant decrease in β PF with age which was more localized to the central-parietal sensors (generally found in depression and other psychological disorders’ patients in open-eye condition; Roohi-Azizi et al., 2017). In the BW measure, only β BW was found to increase significantly with age which indicates higher variability in β frequency (Fig. 7). This increase was mostly observed in the central and temporal sensors. Differences in the regional attenuation of absolute and relative β power within specific high frequency bands may reflect the disparate neuropathologic processes of mild cognitive impairment associated with age, as well as the extent of brain dysfunction. We can further speculate the amount of spread of power in a particular frequency range that increases with age may suggest a state of fractured synchronization in elderly compared with younger adults. Mechanistically, this can be a result of decreased myelination in long-range fibers across aging, which introduces time delays in the resultant dynamical system governing such processes. Time delays are known to introduce phase lags in a group of coupled (and synchronized) oscillators resulting in lowering of synchronization indices such as increased bandwidth.

In conclusion, our results suggest that the age-associated change in aperiodic 1/f activity affects the global information processing and links with speed of information processing, cognitive capacity, precision, and metacognitive awareness (all behavioral measures). In contrast, periodic features; PF and BR of different frequency bands relate to more local processing and selective behavioral measures in VSTM task crucially impacting distinct aspects of memory processing with age. On that account, we suggest that the change observed in local tissue properties with aging is reflected as the global increase in aperiodic 1/f slope. This increase in 1/f slope seems to impact distributed processes of cognition as it alters the self-organized critical functional brain states, whereas oscillatory features mediate localized processing, that is relevant for the specific task (Fig. 12).

An important limitation of our study is that we have only tested aperiodic 1/f slope-offset, and periodic features PF and BR based on VSTM task; therefore, further investigation is warranted relating the RS 1/f slope-offset, PF, BR with the performance in different cognitive tasks (e.g., lexical processing, episodic memory encoding and retrieval, emotion regulation, fluid and crystallized intelligence). Additionally, we have also not looked at these three measures trial-wise instead related resting state brain dynamics (periodic and aperiodic) with behavioral responses from the same participants. Another major limitation was posed by the Cam-CAN dataset, because of the presence of harmonics of lower frequencies in higher frequencies, we were not able to systematically tease apart the effect of 1/f activity on γ-frequency band. From a recent study employing visual steady state response task it has been shown γ band power systematically weakens with age which may have a crucial impact on attentional processing (Murty et al., 2020). Lastly, we still do not know which sources are responsible for 1/f baseline shift and is currently investigated in a separate future work, where source reconstruction and applying computational modeling on the source level data give us mechanistic understanding about the generative processes.

Despite these limitations, we think there is no loss of generality by focusing on visual short-term working memory processing task alone as age-associated change in aperiodic 1/f activity is pervasively present in all goal directed tasks and same for oscillatory changes quantified by PF and BR. Therefore, all these three normative measures proposed in this study together can track vast majority of alterations associated with healthy and atypical neurodevelopment and healthy and pathologic aging conditions under a variety of task settings which is important for developing non-invasive biomarker in future clinical applications.

## Synthesis

Reviewing Editor: Satu Palva, University of Helsinki Decisions are customarily a result of the Reviewing Editor and the peer reviewers coming together and discussing their recommendations until a consensus is reached. When revisions are invited, a fact-based synthesis statement explaining their decision and outlining what is needed to prepare a revision will be listed below. The following reviewer(s) agreed to reveal their identity: Julien Lagarde.

The authors use a large publicly available magnetoencephalography (MEG) dataset to investigate periodic and aperiodic changes across the adult lifespan. They relate these neurophysiological changes with cognitive processing as measured through a visual short-term memory (VSTM) task adapted from Zhang et al., 2008. The authors report strong replications regarding how certain aperiodic (1/f slope and offset) and periodic (central frequency, power, bandwidth, and band ratios) features change with age. They do this using a relatively new approach for separating these components in the spectral domain. Subsequently, they relate these are-related neural changes to the cognitive measures in the VSTM task. These analyses reveal that aperiodic features tend to reflect more global changes in cognitive capacity, while periodic features have a more task-specific influence on behavior.

Overall the methods are sound, the question interesting, and the results are clear.

Figures:

Figure 12 primarily adds confusion for the reader. The main source of confusion stems from how little is said about this figure: it is only referenced once in the discussion, even though it appears to be presenting additional analyses, and its figure legend is sparse, providing little in the way of details. More specifically, the figure looks like it is performing a mediation analysis due to the structure of the task-specific features section. If this is the case, the authors need to expand on this much more: how was the mediation analysis done, what do we learn from it, and so on.

There were also a few minor typos within the figures as well as with referencing the figures. In Figure 4A, the age group labels are not consistent (the oldest group is referenced as “OE” in some plots and “OA” in other plots). Additionally, when referencing Figure 9 in the text, I believe the following typos occurred: Figure 9B should be 9C, Figure 9C should be 9D, and Figure 9D should be 9B. Finally, the first plot in Figure 10 does not have a dashed line representing the high load condition.

Metacognitive task comment:

The metacognitive VSTM task feature may need more explanation in the methods. On first reading, it was not immediately clear what it was measuring or how. Because this component is referenced differently throughout the paper, it adds some confusion for the reader (Mean Uncertainty and Metacognitive Awareness). Additionally, the y-axes in Figure 9 and 10 create more confusion about this measure due to the units (deg) and range of values (0-50). It seems that the y-axes for the precision and mean uncertainty plots are switched and/or the units are incorrect?

High vs Low load comment:

The authors break down the VSTM task into “high load” (set size 4) and “low load” (set size 2) groups to report cognitive decline with age. Although they report what these conditions mean in the Figure 9 and 10 legends, they do not include a description of this categorical separation in the methods. Why not use all set size data? The authors also report the findings for high and low load in all behavioral features except VSTM capacity (k) and do not explain why. Overall, it is not clear why this categorical separation for cognitive load is necessary for this analysis.

Data and Code Use

Where we can find the data and Code used in the study?

Other comments:

The effect in 9C in the low load condition seems very small: (k) for the 20 year olds seems very similar to that of the 80 year olds.

Related to this, how (and why?) is capacity (k) calculated for each set size? Capacity is usually estimated using performance across set sizes.

The authors report many results about bandwidth, but what do we make of that? What does spectral bandwidth mean? Or tell us about aging?

There are some typos and inconsistencies in the paper. For instance, there is not a consistent way of spelling “aging” (sometimes spelled “ageing”), and some capitalized words that may not need to be capitalized (i.e. “Global”).

The naming convention for the age groups is a bit unintuitive for the reader. Middle Elderly sounds like an older age group than Middle Late.

There are multiple acronyms here that are either never defined, or defined after they are first presented. For example: “VSTM” and “RS” are never explicitly defined, and “TAR” is only defined after the first mention.

The authors may want to cite some additional papers. More specifically, when discussing the change in underlying excitation-inhibition (E/I) balance, this is directly related to Gao et al., 2017, which I believe is the only paper that attempts to directly test this. In addition, the authors reference Gazzaley et al., 2005 in support of ageing-related 1/f changes in attention, but that paper is an fMRI study that doesn’t mention 1/f at all.

abstract: acronym “VSTM task.” not defined in it; the task is explained page 5

It is said that 280 Ss from 700: how the selection was made?

More details are required to explain the estimation power spectra p 6 line 49 with Welch’s method: First, the estimation divides the signal into η segments (creating epochs) with some overlap. Subsequently, the epoched signal further windowed using a window function.” Please develop please, which window function was used?

Equation 1 is not enought detailed, what is the average ? 1/N (sum(...))

p 8 eq 4 line 76: what is (captial) F denoting?

How was the Foof model fitting performed? It is said using Fooof toolbox, please make this explicit,

is it least square, a native function of matlab, etc.

## Author Response

Synthesis of Reviews:

Computational Neuroscience Model Code Accessibility Comments for Author (Required): The novel code should be added as extended data or similar and a comment on how to find the code should be added to the manuscript.

Thank you for your suggestion. In the revised version, we have now added the code accessibility statement. However, we have redacted this link during our first submission intentionally to adhere to eNeuro double blind review policy. Please see our response below to comment 4.

Significance Statement Comments for Author (Required):

N/A

Comments on the Visual Abstract for Author (Required):

N/A

Synthesis Statement for Author (Required):

The authors use a large publicly available magnetoencephalography (MEG) dataset to investigate periodic and aperiodic changes across the adult lifespan. They relate these neurophysiological changes with cognitive processing as measured through a visual short-term memory (VSTM) task adapted from Zhang et al., 2008. The authors report strong replications regarding how certain aperiodic (1/f slope and offset) and periodic (central frequency, power, bandwidth, and band ratios) features change with age. They do this using a relatively new approach for separating these components in the spectral domain. Subsequently, they relate these are-related neural changes to the cognitive measures in the VSTM task. These analyses reveal that aperiodic features tend to reflect more global changes in cognitive capacity, while periodic features have a more task-specific influence on behaviour. Overall the methods are sound, the question interesting, and the results are clear.

We would like to thank the Editors and Reviewers for their positive evaluations, constructive comments, and for the opportunity to submit a revised manuscript. We feel that the comments and suggestions improved the quality of our manuscript. We addressed all questions and suggestions of the Reviewers in a point-by-point fashion below and highlighted the corresponding changes in the manuscript in red.

1. Figures Comment:

(a) Figure 12 primarily adds confusion for the reader. The main source of confusion stems from how little is said about this figure: it is only referenced once in the discussion, even though it appears to be presenting additional analyses, and its figure legend is sparse, providing little in the way of details. More specifically, the figure looks like it is performing a mediation analysis due to the structure of the task-specific features section. If this is the case, the authors need to expand on this much more: how was the mediation analysis done, what do we learn from it, and so on.

We agree that very little is said about figure 12 including figure legend. Thank you for pointing this. In the revised submission, we have included the details in the figure legend (see below the excerpt). No additional analysis is being presented/performed in Figure 12. It represents the summary and conclusion of the main results. We have also cited the figure in the relevant context.

"Figure 12. Summary: Aperiodic 1/f activity affects global processing and oscillatory features.

Aperiodic 1/f slope with age affects global processing and index behaviour in all cognitive domain: Aperiodic 1/f slope increases globally which is reflected in performance in different cognitive tasks which includes VSTM task precision, cognitive capacity and reaction time and in other cognitive tasks (N900 lexical prediction, working memory, grammer learning) reported previously. Global changes in 1/f also affects processing uncertainty and metacognitive awareness during VSTM task.

Aperiodic 1/f slope Affects oscillatory features and index task specific behaviour: Aperiodic 1/f slope also significantly affects oscillatory features peak frequency, power and band ratio, which are more task specific in nature. Alpha band power, Theta/Alpha (θ/α), and Alpha/Beta (α/β) band ratios index precision, reaction time and cognitive capacity when age is regressed out. R2 represents the Goodness of fit with different behavioural measures treating age and oscillatory features as explanatory variables. Figure also depicts how age and oscillatory features Alpha and Theta band power, Alpha Peak Frequency, Alpha/Beta (α/β) and Theta/Alpha (θ/α) band ratios are correlated with each other. Different colours of line represent the correlation value.”

(b) There were also a few minor typos within the figures as well as with referencing the figures. In Figure 4A, the age group labels are not consistent (the oldest group is referenced as “OE” in some plots and “OA” in other plots).

Additionally, when referencing Figure 9 in the text, I believe the following typos occurred: Figure 9B should be 9C, Figure 9C should be 9D, and Figure 9D should be 9B.

Finally, the first plot in Figure 10 does not have a dashed line representing the high load condition.

Thank you for pointing this out. We have now carefully addressed all the minor typos in the manuscript. The oldest group is now referenced as “OA” throughout the manuscript and in all the plots. Typos present in the previously submitted manuscript while referencing Figure 9 also appropriately referenced in the main text. In Figure 10, we have now added a linear fit for the high load condition. It was not shown in the earlier version as the regression model did not find any significant linear or second order polynomial fit to the scatter data for the high load condition.

2. Metacognitive task comment:

(a) The metacognitive VSTM task feature may need more explanation in the methods. On first reading, it was not immediately clear what it was measuring or how. Because this component is referenced differently throughout the paper, it adds some confusion for the reader (Mean Uncertainty and Metacognitive Awareness).

Thank you for suggesting this. We have added a detailed explanation in the method section regarding how metacognitive awareness of participants was estimated using mean uncertainty measure. Please see below an excerpt of what we have added now in the revised manuscript (Lines 141-146).

"Subjects indicated their uncertainty in their choice of colour by the length of time they touched the wheel: As they held their finger down, white confidence intervals spread out around the selected point indicating greater uncertainty about their selection. To assess metacognitive awareness, the angular width of the reported confidence intervals provided a trial-by-trial measure of subjective uncertainty. To summarize overall uncertainty for each individual and condition, the mean was taken across trials. Participants with smaller values thus reported more confidence in their responses.”

(b) The y-axes in Figures 9 and 10 create more confusion about this measure due to the units (degree) and range of values (0-50). It seems that the y-axes for the precision and mean uncertainty plots are switched and/or the units are incorrect?

Thank you for pointing this out. We have now rectified the typos in the figure. The unit for precision is inverse of ‘deg-1’ whereas the unit for mean uncertainty is ‘deg’. Mean uncertainty was calculated by estimating the angular width of the confidence interval (in degrees). The longer the participants held their finger down, the more the angular width and it was within the range of 0-50 degrees.

3. High vs Low load Comment:

(a) The authors break down the VSTM task into “high load” (set size 4) and “low load” (set size 2) groups to report cognitive decline with age. Although they report what these conditions mean in the Figure 9 and 10 legends, they do not include a description of this categorical separation in the methods.

(b) Why not use all set size data? The authors also report the findings for high and low load in all behavioural features except VSTM capacity (k) and do not explain why. Overall, it is not clear why this categorical separation for cognitive load is necessary for this analysis.

Thank you for these suggestions.

(a) We agree and accordingly we have added this categorical separation in the methods. Please see below an excerpt of what we have added in the revised manuscript in lines 237-240.

"To clearly delineate the effects of periodic and aperiodic features on VSTM features it was necessary to illustrate the effect in different load conditions. Only set size 2 and set size 4 is reported in the main manuscript where we have categorised the set sizes in two load conditions: set size 2 as a low load condition and set size 4 as a high load condition. We have provided the findings for behavioural features of other set sizes as Extended data.”

(b) We did not include all the set sizes for the main analyses as the focus was to look at the cognitive decline with age which can be seen in set size 2 sparsely and clearly in set size 4. To clearly illustrate and delineate the effects of periodic and aperiodic features, the categorical separation of high and low load was done. However, we have also included all the results for all the participants for all the set sizes as extended data.

In the previously submitted version of the manuscript, we have already reported the findings for high and low load in all behavioural features including VSTM capacity (k).

We have reported the findings for high and low load in VSTM capacity (k) in line 390:

"VSTM capacity was found to decrease with age (Low load (r) = -0.81 p<0.001, High load (r) = -0.87, p <0.001)"

4. Data and Code Use

MEG Data and VSTM data used for this study can be obtained from the Cam-CAN repository (available at http://www.mrc-cbu.cam.ac.uk/datasets/camcan/) (Taylor et al., 2007; Shafto et al., 2014).

The codes for all the analysis carried out in this paper is freely available to download from https://drive.google.com/drive/folders/1__74tFI1_VnHaV_-kJ46VAGMGiI04T2i?usp=sharing

5. Other comments:

(a) The effect in 9C in the low load condition seems very small: (k) for the 20 year olds seems very similar to that of the 80 year olds.

We agree that the effect in low load condition for capacity as small as set size 1 and 2 is cognitively less demanding for the elderly and therefore, less affected due to aging. While older participants could still encode 2 items in their short term memory they exhibit lower precision during memory recall and the difference is small but significant.

(b) Related to above, how (and why?) is capacity (k) calculated for each set size? Capacity is usually estimated using performance across set sizes.

Thank you for your comments. We have now addressed this and accordingly made changes to the revised manuscript.

Capacity is calculated by fitting the error distribution with a uniform distribution which is represented by a single parameter, Pm, which is the probability that the probed item was present in memory at the time of the probe. ‘K’ for each set size is calculated by multiplying the memory load by ‘Pm’.

We have added a detailed explanation for how each behavioural feature is being estimated. Please see below an excerpt of what we have added in the revised manuscript in lines 126-141.

"For each set size (memory load), the behavioural measures were estimated by fitting the error distribution with a mixture model of von-mises and uniform distributions, proposed by Zang & luck (2008) and modified by Bays and Husain (2008). For detailed analysis refer to Zhang et al., 2008; Mitchell et al., (2018). In brief, as a model-free index of performance, the response error-the angular difference between the target color presented and the color reported-was calculated. This model-free index cannot be used to distinguish errors due to imprecise memory of an item, from errors due to reporting the wrong item, or guessing when an item is not kept in memory at all. To estimate these, Maximum likelihood estimation was used to decompose the data from each subject into three parameters that represent a mixture of a uniform distribution of errors and a von Mises distribution of errors. The von Mises distribution is the circular analogue of the gaussian distribution and was used because the tested colour space was circular. The uniform distribution was represented by a single parameter, Pm, which is the probability that the probed item was present in memory at the time of the probe. ‘K’ for each set size is calculated by multiplying the memory load by ‘Pm’. The von Mises distribution was represented by two parameters, its mean (μ) and its standard deviation (s.d.). μ reflects any systematic shift of the distribution away from the original colour value. The ‘precision’ of each item held in memory is reported as the reciprocal of the standard deviation of the fitted von-Mises distribution. “

(c) The authors report many results about bandwidth, but what do we make of that? What does spectral bandwidth mean? Or tell us about aging?

We completely agree that bandwidth requires more explanation and accordingly we have elaborated further in the revised manuscript.

Bandwidth is one of the peak parameters used in this study. It is a parameter that quantifies the strength of signal at a specific frequency. It defines the width of the peak which indicates the amount of spread of power in a particular frequency range that increases with age suggesting integration of information over a shorter time scale mediated by relatively slower frequencies. This is particularly true as we know from previous studies that communication through coherence and long range inter sensor synchronization changes with aging in a frequency dependent manner (Donoghue et al., 2020, Sahoo et al., 2020). For EEG/ MEG signal however where peak frequencies represent the centre frequency of synchronization across a large neural population or mean field activity, bandwidth may stem of a diffused phase synchronization. For faster frequencies, hence, the bandwidth becomes wider and in contrast for slower frequencies it becomes narrower. From the perspective of ageing, a higher bandwidth can represent a state of fractured synchronization in elderly compared to younger adults. Mechanistically, this can be a result of decreased myelination in long-range fibres across ageing (Grydeland et al., 2019) which introduces time-delays in the resultant dynamical system governing such processes (Pajevic et al., 2014). Time delays are known to introduce phase lags in a group of coupled (and synchronized) oscillators resulting in lowering of synchronization indices such as increased band width (Pajevic et al., 2014).

We have added the following lines in the revised manuscript.

Line 185-188:

"Larger bandwidth in given frequency band indicates the deviation of power from the baseline and the spread across a wider frequency range. This global spread in power across frequency band further quantifies the strength of a signal at a specific frequency and allows for information transfer across wider frequency range.”

Line 330-331:

"Increase in beta bandwidth indicates that the beta power tends to be more distributed within the frequency band as we age.”

Line 540-550 in the discussion section we have added the following

"In the bandwidth measure, only Beta bandwidth was found to increase significantly with age which indicates higher variability in beta frequency (Fig. 7). This increase was mostly observed in the central and temporal sensors. Differences in the regional attenuation of absolute and relative beta power within specific high frequency bands may reflect the disparate neuropathologic processes of mild cognitive impairment associated with age, as well as the extent of brain dysfunction. We can further speculate the amount of spread of power in a particular frequency range that increases with age may suggest a state of fractured synchronization in elderly compared to younger adults. Mechanistically, this can be a result of decreased myelination in long-range fibres across ageing (which introduces time-delays in the resultant dynamical system governing such processes. Time delays are known to introduce phase lags in a group of coupled (and synchronized) oscillators resulting in lowering of synchronization indices such as increased band width.”

(d) There are some typos and inconsistencies in the paper. For instance, there is not a consistent way of spelling “aging” (sometimes spelled “ageing”), and some capitalized words that may not need to be capitalized (i.e., “Global”).

We have corrected all the inconsistencies found in the paper. Aging is now being referred as “ageing” throughout the paper.

(e) The naming convention for the age groups is a bit unintuitive for the reader. Middle Elderly sounds like an older age group than Middle Late.

In defining age categories, we have followed extant literature and named the categories accordingly which allow us to have a fair comparison with the previous findings indexing important milestones of aging. Earlier studies have done similar age wise stratifications (Chan et al., 2014; Sahoo et al., 2020). In order to be consistent with the existing studies, we have followed the same conventions.

(f) There are multiple acronyms here that are either never defined or defined after they are first presented. For example: “VSTM” and “RS” are never explicitly defined, and “TAR” is only defined after the first mention.

Thank you for pointing this out. We completely agree that the acronyms were defined at times after their first mention. In the revised manuscript, we have now corrected this and defined these acronyms before their first use.

VSTM: Visual short-term memory

RS: Resting State

TAR: Theta/Alpha ratio

(g) The authors may want to cite some additional papers. More specifically, when discussing the change in underlying excitation-inhibition (E/I) balance, this is directly related to Gao et al., 2017, which I believe is the only paper that attempts to directly test this. In addition, the authors reference Gazzaley et al., 2005 in support of ageing-related 1/f changes in attention, but that paper is an fMRI study that doesn’t mention 1/f at all.

We agree that Gao et al., 2017 is one of the important papers to be cited in the context of E/I balance and accordingly we have cited and referenced it in the relevant context in Line 48 in the revised manuscript.

Thank you for pointing this out. Gazzaley et al., 2005 was supposed to be cited in the context of cognitive decline with age. Please see below an excerpt of the correction made in lines 41-45.

"As we age, we are faced with the likelihood that our cognitive faculties will decline for example, ascertain memory (Nyberg et al, 2012), shift of sustained attention (Gazzaley et al., 2005), and processing speed (Salthouse et al., 2010). There were recent attempts to relate 1/f activity - where it is being considered as a marker of “neural noise"- with N900 lexical prediction (Dave et al., 2018), working memory (Voytek et al., 2020) and grammar learning (Cross et al., 2020).”

(h) abstract: acronym “VSTM task.” not defined in it; the task is explained page 5

In the revised manuscript, we have defined the acronym VSTM in the abstract.

(i) It is said that 280 Ss from 700: how was the selection made?

We randomly selected 280 participants out of all the 700 participants from Cam-CAN, carried out the analysis and iterated this 10 times to remove any sampling bias but for the main results we worked with one group of 280 participants.

(j) More details are required to explain the estimation power spectra p 6 line 49 with Welch’s method: First, the estimation divides the signal into η segments (creating epochs) with some overlap. Subsequently, the epoched signal further windowed using a window function.” which window function was used?

We agree that the way we had written this in the previous version of our manuscript was quite confusing. We have now removed that paragraph and explicitly added which window was used in Line 170-173.

"Each sensor’s c^’ s time series xc(t) was further divided into segments of 20s (epochs) without any overlap. Spectrum was estimated for each segment after multiplying the time series segment with a Hanning window. We estimated a global spectrum, representative of each subject i.e., SI(f) by taking a grand average of spectrums across all 102 magnetometer channels.”

(k) Equation 1 is not detailed enough, what is the average? 1/N (sum(...))

Thank you for your suggestion.

To be more precise and clear we have modified this sentence in the revised manuscript and added more details to describe this equation (Line 171-173):

"We estimated a global spectrum, representative of each subject i.e., SI(f) by taking a grand average of spectrums across all 102 magnetometer channels.”

〖 S〗_I(f)=∑_c▒〖s_I (c,f) 〗

(l) p 8 eq 4 line 76: what is (captial) F denoting?

‘F’ denotes the frequency vector. This has already been defined in Eq. (3). Please see below

G_n=a*e^((- (F-C)^2/〖2w〗^2 ) )

Where ‘a’ denotes the amplitude, ‘c’ denotes the central frequency, ‘w’ denotes the bandwidth of the Gaussian. ‘F’ is the frequency vector.

Since this has already been defined and introduced in line 195-196 in equation 3, we have not considered defining ‘F’ again in equation 4 as this is redundant.

(m) How was the Fooof model fitting performed? It is said using Fooof toolbox, please make this explicit,

is it least square, a native function of matlab etc.

Thank you for your suggestion. We have added the following details in the revised version of our manuscript.

We have followed your suggestion and provided detailed descriptions of how FOOOF model fitting was performed in section 2.4.2. Full description of the model can be found somewhere else (Donoghue et al., 2020).

Following is an excerpt from what we have added in the revised manuscript Lines 202-209.

‘F’ is the frequency vector. The Fooof model described in equation 2 fits the power of a given sensor signal by estimating a linear function ‘L’ for the aperiodic component of the signal and each oscillatory contribution ‘Gn’ is modelled as Gaussian peaks. Moreover, estimated power was fitted across the entire frequency range of 1 to 45 Hz by as no knee was expected in the MEG recordings across 1-50 Hz frequency range (Miller et al., 2009). The number of oscillatory components is determined from the data, with the option to set a maximum number of components as a parameter. The general model assumption here is that oscillatory and aperiodic processes are distinct and separable.

The model was fitted for individual subject and output parameters were averaged across subjects for each group (Figure. 3(A)). The settings for the algorithm were set as: (1) peak_width_limits = [0.5, 12]; (2) min_peak_height = 0; (3) max_n_peaks = 12; (4) peak_threshold = 2; (5) aperiodic_mode = “fixed"; and (6) verbos = ‘True’.

References

Donoghue, T., Dominguez, J., & Voytek, B. (2020). Electrophysiological frequency band ratio measures conflate periodic and aperiodic neural activity. Eneuro, 7(6).

Gao, R., Peterson, E. J., & Voytek, B. (2017). Inferring synaptic excitation/inhibition balance from field potentials. Neuroimage, 158, 70-78.

Gazzaley A, Cooney JW, Rissman J, D’Esposito M (2005) Top-down suppression deficit underlies working memory impairment in normal ageing. Nat Neurosci 8:1298-1300.

Sahoo, B., Pathak, A., Deco, G., Banerjee, A., & Roy, D. (2020). Lifespan associated global patterns of coherent neural communication. NeuroImage, 116824.

Shafto, M. A., Tyler, L. K., Dixon, M., Taylor, J. R., Rowe, J. B., Cusack, R., ... & Henson, R. N. (2014). The Cambridge Centre for Ageing and Neuroscience (Cam-CAN) study protocol: a cross-sectional, lifespan, multidisciplinary examination of healthy cognitive ageing. BMC neurology, 14(1), 204.

Taylor, J. R., Williams, N., Cusack, R., Auer, T., Shafto, M. A., Dixon, M., ... & Henson, R. N. (2017). The Cambridge Centre for Ageing and Neuroscience (Cam-CAN) data repository: Structural and functional MRI, MEG, and cognitive data from a cross-sectional adult lifespan sample. Neuroimage, 144, 262-269.

Zhang, W., & Luck, S. J. (2008). Discrete fixed-resolution representations in visual working memory. Nature, 453(7192), 233-235.

Bays, P. M., & Husain, M. (2008). Dynamic shifts of limited working memory resources in human vision. Science, 321(5890), 851-854.

Grydeland, H., Vértes, P. E., Váša, F., Romero-Garcia, R., Whitaker, K., Alexander-Bloch, A. F., ... & Bullmore, E. T. (2019). Waves of maturation and senescence in micro-structural MRI markers of human cortical myelination over the lifespan. Cerebral Cortex, 29(3), 1369-1381.

Pajevic, S., Basser, P. J., & Fields, R. D. (2014). Role of myelin plasticity in oscillations and synchrony of neuronal activity. Neuroscience, 276, 135-147.
