# Supplementary material for: Aperiodic and Periodic Components of Ongoing Oscillatory Brain Dynamics Link Distinct Functional Aspects of Cognition across Adult Lifespan
Source: eNeuro. 2021 Oct 15;8(5):ENEURO.0224-21.2021. doi: 10.1523/ENEURO.0224-21.2021 (PMC8547598; doi:10.1523/ENEURO.0224-21.2021)
Supplement: Extended Data Table 8-1 — Regression table for global frequency BRs with age. F value, β coefficient, goodness of fit, and significance of the model are reported. Download Table 8-1, DOC file. [file enu-eN-NWR-0224-21-s20.doc]

**Table 8-1**

| Explanatory Variable | Response Variable | F-value | Beta1 | p-value | R^2^ |
| --- | --- | --- | --- | --- | --- |
| Age | 𝛼/𝛽 PW | 77 | -0.018116 | 1.44e-06 | 0.86 |
|  | 𝛼/𝛽 CF | 8.7 | -0.005913 | 0.0054 | 0.62 |
|  | 𝜃/𝛽 BW | 5.31 | -0.001542 | 0.03 | 0.3 |
|  | 𝜃/𝛼 PW | 7.2 | +0.00576 | 0.02 | 0.39 |
