# Supplementary material for: Aperiodic and Periodic Components of Ongoing Oscillatory Brain Dynamics Link Distinct Functional Aspects of Cognition across Adult Lifespan
Source: eNeuro. 2021 Oct 15;8(5):ENEURO.0224-21.2021. doi: 10.1523/ENEURO.0224-21.2021 (PMC8547598; doi:10.1523/ENEURO.0224-21.2021)
Supplement: Extended Data Table 9-1 — Regression table for VSTM measures with age. F value, β coefficient, goodness of fit, and significance of the model are reported. Download Table 9-1, DOC file. [file enu-eN-NWR-0224-21-s21.doc]

# Table 9-1

| Explanatory Variable | Response Variable | | F-value | Beta1 | p-value | R^2^ |
| --- | --- | --- | --- | --- | --- | --- |
| Age | Behavioral Measure | Load (Set-size) |  |  |  |  |
|  | k (capacity) | 4 | 36.2 | -0.02274 | 6.02e ^-05^ | 0.75 |
|  |  | 2 | 30.2 | -0.00372 | 0.00014 | 0.7 |
|  | RT | 4 | 5.2 | +4.5738 | 4.00e-02 | 0.3 |
|  |  | 2 | 3.89 | +4.09 | 5.00e-03 | 0.26 |
|  | d (uncertainty) | 4 | 3.9 | +-0.17 | 0.05 | 0.25 |
|  |  | 2 | 4.89 | -0.10297 | 0.04 | 0.29 |
|  | Precision | 4 | 1.85 | -0.00867 | 0.02 | 0.39 |
|  |  | 2 | 30.2 | -0.0018 | 1.38e-04 | 0.7 |
