# Supplementary material for: Aperiodic and Periodic Components of Ongoing Oscillatory Brain Dynamics Link Distinct Functional Aspects of Cognition across Adult Lifespan
Source: eNeuro. 2021 Oct 15;8(5):ENEURO.0224-21.2021. doi: 10.1523/ENEURO.0224-21.2021 (PMC8547598; doi:10.1523/ENEURO.0224-21.2021)
Supplement: Extended Data Table 10-1 — Regression table for VSTM measures with aperiodic slope. F value, β coefficient, goodness of fit, and significance of the model are reported. Download Table 10-1, DOC file. [file enu-eN-NWR-0224-21-s22.doc]

# Table 10-1

| Explanatory Variable | Response Variable | | F-value | Beta1 | p-value | R^2^ |
| --- | --- | --- | --- | --- | --- | --- |
| Aperiodic 1/f Slope | Behavioral Measure | Load (Set-size) |  |  |  |  |
|  | k (capacity) | 4 | 25.9 | -6.2486 | 0.0003 | 0.702 |
|  |  | 2 | 2.98 | -0.75038 | 0.112 | 0.2 |
|  | RT | 4 | 10.6 | +2666.3 | 0.0076 | 0.49 |
|  |  | 2 | 9.59 | +2336.3 | 0.0102 | 0.466 |
|  | d (uncertainty) | 4 | 0.25 | -46.1 | 0.02 | 0.35 |
|  |  | 2 | 5.5 | -48.817 | 0.0388 | 0.33 |
|  | Precision | 4 | 1.52 | -0.10677 | 0.24 | 0.11 |
|  |  | 2 | 36.9 | -0.44753 | 5.52e-05 | 0.75 |
