# Supplementary material for: Aperiodic and Periodic Components of Ongoing Oscillatory Brain Dynamics Link Distinct Functional Aspects of Cognition across Adult Lifespan
Source: eNeuro. 2021 Oct 15;8(5):ENEURO.0224-21.2021. doi: 10.1523/ENEURO.0224-21.2021 (PMC8547598; doi:10.1523/ENEURO.0224-21.2021)
Supplement: Extended Data Table 10-2 — Regression table for VSTM measures with aperiodic 1/f offset. F value, β coefficient, goodness of fit, and significance of the model are reported. Download Table 10-2, DOC file. [file enu-eN-NWR-0224-21-s23.doc]

**Table 10-2**

| Explanatory Variable | Response Variable | | F-value | Beta1 | p-value | R^2^ |
| --- | --- | --- | --- | --- | --- | --- |
| Aperiodic  1/f Offset | Behavioral Measure | Load (Set-size) |  |  |  |  |
|  | k (capacity) | 4 | 2.06 | +2.3507 | 0.179 | 0.158 |
|  |  | 2 | 1.53 | +0.4515 | 0.241 | 0.122 |
|  | RT | 4 | 8.26 | -1979.2 | 0.01 | 0.429 |
|  |  | 2 | 10.1 | -1880.9 | 0.008 | 0.47 |
|  | d (uncertainty) | 4 | 0.168 | -14.374 | 0.69 | 0.015 |
|  |  | 2 | 0.559 | -14.762 | 0.47 | 0.04 |
|  | Precision | 4 | 5.2e-05 | -0.0009 | 0.99 | 4.7e-06 |
|  |  | 2 | 0.658 | -0.1406 | 0.52 | 0.05 |
