# Supplementary material for: Aperiodic and Periodic Components of Ongoing Oscillatory Brain Dynamics Link Distinct Functional Aspects of Cognition across Adult Lifespan
Source: eNeuro. 2021 Oct 15;8(5):ENEURO.0224-21.2021. doi: 10.1523/ENEURO.0224-21.2021 (PMC8547598; doi:10.1523/ENEURO.0224-21.2021)
Supplement: Extended Data Table 11-1 — Regression table for specific oscillatory features with VSTM measures. F value, β coefficient, goodness of fit, and significance of the model are reported. Download Table 11-1, DOC file. [file enu-eN-NWR-0224-21-s24.doc]

**Table 11-1**

| Explanatory Variable | Response Variable | | F-value | Beta1 | p-value | R^2^ |
| --- | --- | --- | --- | --- | --- | --- |
| Alpha CF | Behavioral Measure | Load (Set-size) |  |  |  |  |
|  | RT | 4 | 7.89 | -352.41 | 0.0158 | 0.39 |
|  |  | 2 | 9.08 | -340.82 | 0.0108 | 0.43 |
| Alpha PW | Precision | 4 | 7.4 | +0.1761 | 0.0186 | 0.38 |
|  |  | 2 | 8.89 | +0.2807 | 0.0115 | 0.42 |
| Theta PW | k(capacity) | 4 | 9.46 | -1.8862 | 0.0106 | 0.46 |
|  |  | 2 | 12.5 | -0.63163 | 0.0046 | 0.53 |
