# Supplementary material for: Aperiodic and Periodic Components of Ongoing Oscillatory Brain Dynamics Link Distinct Functional Aspects of Cognition across Adult Lifespan
Source: eNeuro. 2021 Oct 15;8(5):ENEURO.0224-21.2021. doi: 10.1523/ENEURO.0224-21.2021 (PMC8547598; doi:10.1523/ENEURO.0224-21.2021)
Supplement: Extended Data Table 11-2 — Regression table for α/β CF with VSTM measures. F value, β coefficient, goodness of fit, and significance of the model are reported. Download Table 11-2, DOC file. [file enu-eN-NWR-0224-21-s25.doc]

**Table 11-2**

| Explanatory Variable | Response Variable | | F-value | Beta1 | p-value | R^2^ |
| --- | --- | --- | --- | --- | --- | --- |
| 𝛼/𝛽 CF | Behavioral Measure | Load (Set-size) |  |  |  |  |
|  | k (capacity) | 4 | 3.09 | -6.3816 | 0.1 | 0.2 |
|  |  | 2 | 0.63 | +0.6699 | 0.44 | 0.05 |
|  | RT | 4 | 0.8 | -1510.3 | 0.38 | 0.06 |
|  |  | 2 | 1.96 | -2071.3 | 0.18 | 0.14 |
|  | d (uncertainty) | 4 | 0.58 | -53.371 | 0.46 | 0.04 |
|  |  | 2 | 0.2 | +20.287 | 0.61 | 0.02 |
|  | Precision | 4 | 6.57 | -0.7578 | 0.02 | 0.35 |
|  |  | 2 | 6.33 | +25.304 | 0.01 | 0.53 |
