# Supplementary material for: Aperiodic and Periodic Components of Ongoing Oscillatory Brain Dynamics Link Distinct Functional Aspects of Cognition across Adult Lifespan
Source: eNeuro. 2021 Oct 15;8(5):ENEURO.0224-21.2021. doi: 10.1523/ENEURO.0224-21.2021 (PMC8547598; doi:10.1523/ENEURO.0224-21.2021)
Supplement: Extended Data Table 11-3 — Regression table for α/β Power ratio with VSTM measures. F value, β coefficient, goodness of fit, and significance of the model are reported. Download Table 11-3, DOC file. [file enu-eN-NWR-0224-21-s26.doc]

**Table 11-3**

| Explanatory Variable | Response Variable | | F-value | Beta1 | p-value | R^2^ |
| --- | --- | --- | --- | --- | --- | --- |
| 𝛼/𝛽 PW | Behavioral Measure | Load (Set-size) |  |  |  |  |
|  | k (capacity) | 4 | 15.6 | +0.7898 | 0.06 | 0.56 |
|  |  | 2 | 0.12 | -0.0219 | 0.7 | 0.01 |
|  | RT | 4 | 5.96 | -255.71 | 0.6 | 0.33 |
|  |  | 2 | 3.18 | -188.68 | 0.09 | 0.21 |
|  | d (uncertainty) | 4 | 1.98 | +6.9368 | 0.18 | 0.14 |
|  |  | 2 | 5.06 | +5.6266 | 0.055 | 0.3 |
|  | Precision | 4 | 9.69 | +0.0634 | 0.008 | 0.4 |
|  |  | 2 | 27.3 | +0.1191 | 0.0002 | 0.69 |
