# Supplementary material for: Aperiodic and Periodic Components of Ongoing Oscillatory Brain Dynamics Link Distinct Functional Aspects of Cognition across Adult Lifespan
Source: eNeuro. 2021 Oct 15;8(5):ENEURO.0224-21.2021. doi: 10.1523/ENEURO.0224-21.2021 (PMC8547598; doi:10.1523/ENEURO.0224-21.2021)
Supplement: Extended Data Table 11-4 — Regression table for θ/α power ratio with VSTM measures. F value, β coefficient, goodness of fit, and significance of the model are reported. Download Table 11-4, DOC file. [file enu-eN-NWR-0224-21-s27.doc]

**Table 11-4**

| Explanatory Variable | Response Variable | | F-value | Beta1 | p-value | R^2^ |
| --- | --- | --- | --- | --- | --- | --- |
| 𝜃/𝛼 PW | Behavioral Measure | Load (Set-size) |  |  |  |  |
|  | k (capacity) | 4 | 14.5 | -1.5827 | 0.002 | 0.57 |
|  |  | 2 | 7.96 | -0.3543 | 0.01 | 0.42 |
|  | RT | 4 | 1.25 | +371.2 | 0.288 | 0.10 |
|  |  | 2 | 2.04 | +397.1 | 0.181 | 0.16 |
|  | d (uncertainty) | 4 | 0.25 | +9.321 | 0.652 | 0.01 |
|  |  | 2 | 0.26 | -6.3127 | 0.66 | 0.01 |
|  | Precision | 4 | 0.02 | +0.013 | 0.878 | 0.002 |
|  |  | 2 | 0.15 | -0.0436 | 0.86 | 0.02 |
